# Unravelling Peritoneal Carcinomatosis Using Cross-Sectional Imaging Modalities

**DOI:** 10.3390/diagnostics13132253

**Published:** 2023-07-03

**Authors:** Ana Veron Sanchez, Ilias Bennouna, Nicolas Coquelet, Jorge Cabo Bolado, Inmaculada Pinilla Fernandez, Luis A. Mullor Delgado, Martina Pezzullo, Gabriel Liberale, Maria Gomez Galdon, Maria A. Bali

**Affiliations:** 1 Hospital Universitaire de Bruxelles, Institut Jules Bordet, 1070 Brussels, Belgium; 2 Teleconsult, Milton Keynes MK12 5NE, UK; 3 Hospital Universitario La Paz, 28046 Madrid, Spain; 4 Hospital Universitario Gregorio Marañon, 28009 Madrid, Spain; 5 Hôpital Universitaire de Bruxelles, Hôpital Erasme, 1070 Brussels, Belgium

**Keywords:** peritoneum, carcinomatosis, deposits

## Abstract

Peritoneal carcinomatosis (PC) refers to malignant epithelial cells that spread to the peritoneum, principally from abdominal malignancies. Until recently, PC prognosis has been considered ill-fated, with palliative therapies serving as the only treatment option. New locoregional treatments are changing the outcome of PC, and imaging modalities have a critical role in early diagnosis and disease staging, determining treatment decision making strategies. The aim of this review is to provide a practical approach for detecting and characterizing peritoneal deposits in cross-sectional imaging modalities, taking into account their appearances, including the secondary complications, the anatomical characteristics of the peritoneal cavity, together with the differential diagnosis with other benign and malignant peritoneal conditions. Among the cross-sectional imaging modalities, computed tomography (CT) is widely available and fast; however, magnetic resonance (MR) performs better in terms of sensitivity (92% vs. 68%), due to its higher contrast resolution. The appearance of peritoneal deposits on CT and MR mainly depends on the primary tumour histology; in case of unknown primary tumour (3–5% of cases), their behaviour at imaging may provide insights into the tumour origin. The timepoint of tumour evolution, previous or ongoing treatments, and the peritoneal spaces in which they occur also play an important role in determining the appearance of peritoneal deposits. Thus, knowledge of peritoneal anatomy and fluid circulation is essential in the detection and characterisation of peritoneal deposits. Several benign and malignant conditions show similar imaging features that overlap those of PC, making differential diagnosis challenging. Knowledge of peritoneal anatomy and primary tumour histology is crucial, but one must also consider clinical history, laboratory findings, and previous imaging examinations to achieve a correct diagnosis. In conclusion, to correctly diagnose PC in cross-sectional imaging modalities, knowledge of peritoneal anatomy and peritoneal fluid flow characteristics are mandatory. Peritoneal deposit features reflect the primary tumour characteristics, and this specificity may be helpful in its identification when it is unknown. Moreover, several benign and malignant peritoneal conditions may mimic PC, which need to be considered even in oncologic patients.

## 1. Introduction

The peritoneum is the second most common metastatic location for abdominal tumours, only surpassed by the liver.

Only 10% of the cases of peritoneal carcinomatosis (PC) are related to extra abdominal tumours [1], with breast (41%) and lung cancers (21%) and malignant melanoma (9%) being the most frequent causes [2].

Traditionally, PC implied an ill-fated prognosis and only palliative treatments were applied. However, the introduction of new surgical techniques and regional therapies have changed this scenario, and palliative systemic treatment is no longer the only therapeutic option. A combination of systemic chemotherapy, cytoreductive surgery (CRS), and hyperthermic intraperitoneal chemotherapy (HIPEC) achieves promising results in selected PC cases from colorectal, ovarian, and gastric cancers [3,4,5]. The main purpose of CRS is to remove all visible deposits with curative intent, which may require peritonectomy and visceral resections. Intraperitoneal chemotherapy, its effect enhanced by hyperthermia, is then administered to intensify the dose of chemotherapy delivered to the tumour while controlling systemic toxicity.

Thus, despite its difficulty, the early diagnosis of PC based on imaging findings is essential for disease staging, for the subsequent management of primary tumours and for patient prognosis. Given the aggressiveness of cytoreductive surgery, it is mandatory to adequately select patients in whom the potential benefits will prevail as surgical candidates, distinguishing them from those who should be treated with systemic chemotherapy, either neoadjuvant or palliative. The peritoneal carcinomatosis index (PCI) serves to quantify the extent of the peritoneal involvement and serves as a predictor of incomplete CRS [6].

The aim of this review is to provide a practical approach when encountering a peritoneal carcinomatosis case using cross-sectional imaging, and to establish where to look for deposits, the possible appearances they may show, and the main complications. In the event of no prior known primary tumour, this review also aims to depict the deposits characteristics that may be helpful in suggesting the origin and to describe differential diagnosis.

## 2. Diagnostic Modalities

The gold standard for the assessment of PC is explorative laparoscopy, which is a minimally invasive procedure that allows for direct visualization and biopsy. However, as an initial approach, cross-sectional imaging modalities are preferred, and the most common and widespread imaging technique used is intravenous contrast-enhanced (CE) computed tomography (CT) thanks to its availability, fast acquisition time, and the possibility of multiplanar reconstructions [7,8]. The administration of water density oral contrast may improve the detection of peritoneal deposits, especially those adjacent to the bowel [9]. CE-CT has demonstrated 68% sensitivity and 88% specificity for peritoneal deposits, although their size and location can undermine this performance [10,11]. When the deposit size is less than 10 mm, the sensitivity dramatically descends to 7–28% [6]. Location, especially in the absence of ascites, has an important impact on sensitivity: metastases within the lesser omentum, left subphrenic space, the root of the mesentery, and small bowel serosa are often missed.

Magnetic resonance (MR), including diffusion-weighted imaging (DWI) and contrast-enhanced T1-weighted sequences, is a promising diagnostic modality, as it provides a higher sensitivity (91%) and similar specificity (85%) to CE-CT [9] due to its high contrast resolution, which allows for tumours to be distinguished from surrounding non-tumour tissues.

Several studies have proven that adding DWI to conventional MR increases the detection of peritoneal deposits [10,12,13], even of small size [14]. Tumoral deposits, due to their high cellular content, restrict the diffusion of water and are displayed as high signal intensity lesions [15], which increases their conspicuity. The diffusion properties of tissues can be quantified by calculating the apparent diffusion coefficient (ADC) maps, where low ADC values correspond to restricted diffusion. ADC values of peritoneal metastasis from ovarian tumours have been measured and found to be comparable to ADC values of the primary tumour [16]. Some studies suggest there is a relation between the ADC values of peritoneal deposits and treatment response [17]. If chemotherapy were successful, deposits would show an increase in their ADC value, even before they would decrease in size.

MR may be preferable to CT in the detection of lesions of less than 10 mm, especially within the subphrenic spaces and bowel serosa. Moreover, in the presence of moderate to substantial ascites, MR still performs better than CT [10]; however, on the other hand, the presence of ascites may lead to dielectric artifacts and false positives [18]. The artifacts caused by respiratory and cardiac motion may impair the detection of deposits within subphrenic and perihepatic spaces. Mucinous and deposits with cystic and necrotic changes may increase the ADC value and cause a false negative result [8]. One final limitation is the insufficient experience with MR interpretation among radiologists [19].

A third non-invasive imaging modality available to assess peritoneal carcinomatosis is positron emission tomography fused with CT images (PET-CT), which provides metabolic information of the lesions, based on the measurement of the increased uptake of a radiotracer, mostly a glucose analogue (FDG [18F]-2-deoxy-2-fluoro-D-glucose). It improves the diagnostic performance of CT but shows slightly lower sensitivity (87%) than MR, probably due to its lower spatial resolution, with limited depiction of small nodules. On the other hand, its specificity is slightly higher (92%) [18]. Moreover, FDG-PET-CT provides a whole-body assessment, which is a major advantage in detecting extra-abdominal metastases. However, in addition to the high costs, PET-CT is less available, and it may provide false-negative findings, such as for mucinous tumours [7], and false-positive findings, such as for postoperative abnormalities or infectious or inflammatory conditions. False-negative interpretations may be also due to deposits being obscured by normal bladder or bowel activity.

Radiologists need to be aware of clinical and laboratory findings.

The clinical presentation of PC is variable, nonspecific, and depends on its extension. Patients may present with characteristic symptoms of the primary tumour and nonspecific symptoms. Abdominal pain and ascites occur in 85% of patients [19]. Complications due to PC, such as ureterohydronephrosis, bowel obstruction, and ischemia, could present as acute abdominal pain.

Serum tumour markers are measurable indicators associated with malignancy, produced either by the tumour or by the body in response to the tumour. They do not serve as a main tool for cancer diagnosis but are useful in supporting diagnosis and treatment response [20]. For instance, high carcinoembryonic antigen (CEA) serum levels are found in 60–90% of colorectal carcinoma, 50–80% of pancreatic carcinoma, and 25–50% of gastric and breast carcinoma, and CA-125 is associated with ovarian cancer [21]. Rising levels of tumour markers should raise concerns, as recurrence may form a miliary pattern that is difficult to spot using imaging [22]. Thus, a combination of imaging findings and tumour markers serum levels is used to determine the response to chemotherapy and to detect recurrence if the levels are elevated upon tumour presentation. However, it should be noted that non-malignant entities may show high tumour marker levels and that patients with extensive peritoneal malignant disease recurrence may have normal tumour markers.

How to proceed? CT is a very useful first-line modality [23]. However, in potential candidates for CRS and HIPEC, where imaging assessment regarding patient selection is critical, CT has been found to underestimate the volume of peritoneal disease [24], while MRI-PCI has shown a stronger correlation with surgical PCI [9]. After peritoneal surgery, it is essential to obtain a baseline MR to determine postsurgical appearance in order to avoid overcalling postsurgical changes for recurrence. Postoperative changes will resolve upon following studies and relapse-related findings will progress [25]. Surveillance should also be performed when using MR.

PET-CT should be considered when encountering equivocal imaging findings, when tumour marker levels rise with no identifiable cause upon imaging, and to detect nodal or extra-abdominal metastases.

In our clinical practice, CT images are acquired on the axial plane at the portal venous after intravenous administration of iodine contrast medium, with a flow rate of 3 mL/s. Multiplane reconstruction can be obtained on coronal and sagittal planes. No oral contrast is required. When using a dual-source scanner, virtual, unenhanced images can be generated.

MR images can be obtained using 1.5 T or 3.0 T field-strength MR scanners with a surface-phased array coil covering the abdomen and the pelvis. The acquisition protocol includes coronal and axial T2-weighted images and diffusion-weighted imaging (respiratory-triggered for the upper abdomen and on breath hold for the inferior abdomen) with 3 b values of 0, 150, and 800 s/mm^2^. Following intravenous injection of 0.2 mmol/kg of gadolinium-based contrast agents, fat-suppressed 3D T1-weighted sequences are obtained upon breath hold at the arterial and portal phases covering the upper abdomen and after a 3 min late phase of the whole abdomen.

## 3. Many Places and Many Faces

The term peritoneal carcinomatosis refers to the spread of malignant epithelial cells as tumour deposits to the peritoneum [26].

PC represents a very complex condition at imaging, more than any other metastatic site.

The appearance of the peritoneal deposits is determined not only by the histological characteristics of the primary tumour, the timepoint of the tumour’s evolution, and the treatment [27] but also, and more interestingly, by the peritoneal space in which they occur. Complications secondary to deposits also vary according to the location, depending on the organs involved.

Some anatomic knowledge is required in the quest for deposits. The aim of this review is not to offer a comprehensive description of peritoneal anatomy but to establish sufficient certainty that all locations are thoroughly investigated.

The peritoneal cavity is the virtual space that exists between the parietal and visceral peritoneum [28] and, under normal conditions, contains a small amount of plasma-like fluid.

**Figure 1:**
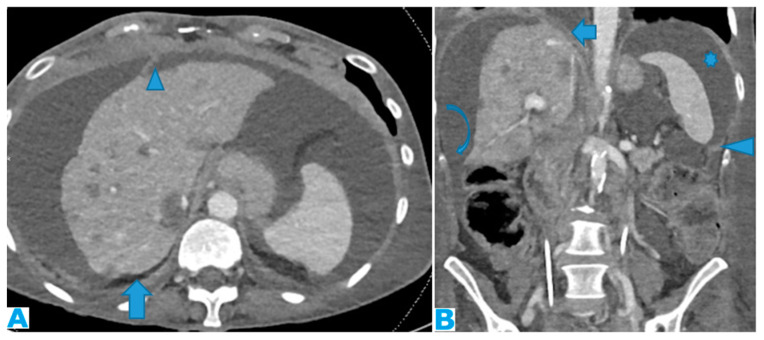
Axial CE-CT (**A**) and coronal MPR (**B**). Massive ascites, helpful to differentiate the peritoneal spaces. (**A**) shows how the posterior margin of the diaphragm (arrow) is not covered by the parietal peritoneum, as it is directly in contact with the retroperitoneal fat. The attachment sites of ligaments on the diaphragmatic surface are not covered by peritoneum; the falciform identified in (**A**) and the phrenicocolic ligaments (arrowhead) in (**B**). Notice in (**B**) how the phrenicocolic ligament partially separates the left parietocolic gutter from the left subphrenic space (*), whereas the right parietocolic gutter fully communicates with the right subphrenic space (curved arrow). The bare area of the liver in (**B**) (arrow) is an area directly attached to the diaphragm by connective tissue and thus not covered by peritoneum, and its diaphragmatic attachment site is not covered either.

**Figure 2:**
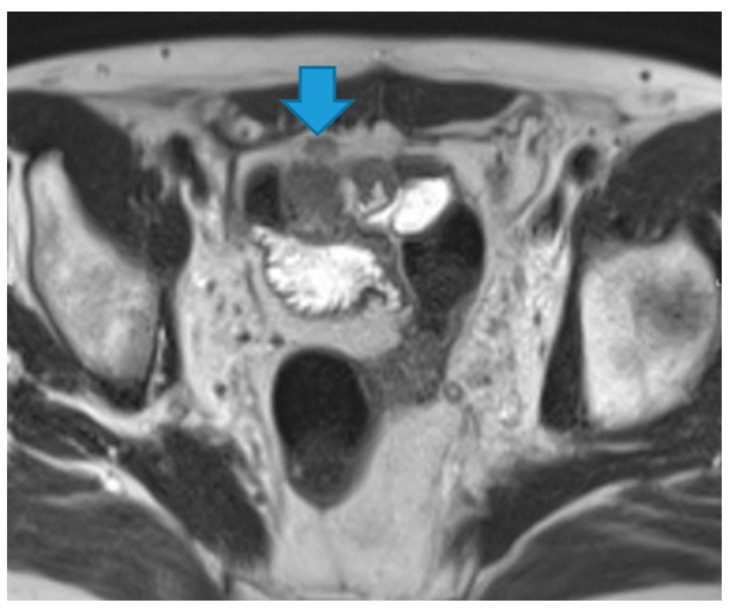
Axial T2WI. PC from neuroendocrine tumour: deposit within the anterior parietal peritoneum (arrow).

**Figure 3:**
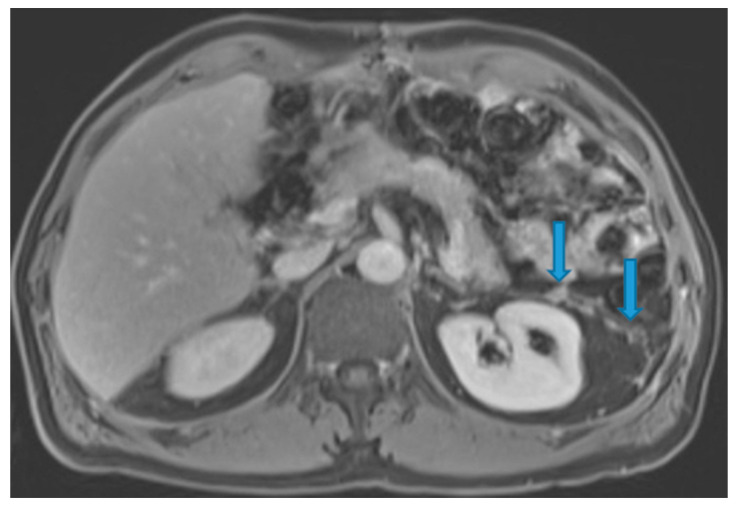
Axial T2WI. PC from neuroendocrine tumour: deposits within the posterior parietal peritoneum (arrows) (which is immediately anterior to the anterior pararenal fascia).

The parietal peritoneum delineates the periphery of the peritoneal cavity:

- Cranially, it covers the diaphragm (except for the bare area of the liver, the insertion of the ligaments, and along its posterior margin, where it is in contact with retroperitoneal fat) (Figure 1).
- Caudally, it descends into the pelvis. Its complex anatomy in this location is shown in more detail later.
- Anterolaterally, it is separated from the abdominal wall by the fat from the preperitoneal space, that is, the space between the peritoneum and the transversalis fascia (Figure 2).
- Posteriorly, it is distanced from the posterior abdominal wall by the retroperitoneal space. It forms the anterior boundary of the retroperitoneal space (Figure 3).

The peritoneum invaginates to fully cover most of the abdominal viscera, anteriorly and posteriorly, becoming the visceral peritoneum. It is organised into a folded disposition as ligaments, folds, and mesenteries to nurture, innervate, and support the intraperitoneal organs, connecting them to the posterior parietal peritoneum.

As a common rule, the abnormal thickening and pathological enhancement of surfaces covered by the peritoneum may be the only initial imaging finding in PC.

**Figure 4:**
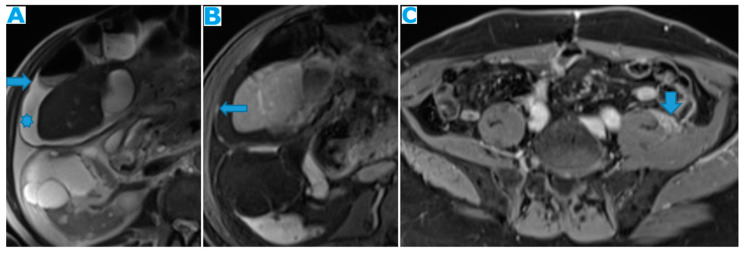
Axial T2WI (**A**) and axial CE portal phase FST1WI (**B**), same patient. PC from colon adenocarcinoma: notice the nodular thickening of the parietal peritoneum due to deposits (arrow). Notice how easy it is to distinguish the parietal peritoneum from the visceral perihepatic peritoneum thanks to the ascites (*). Axial CE portal phase FST1WI (**C**). PC from neuroendocrine tumour: deposits within the posterior parietal peritoneum (arrow). The retroperitoneal space is very thin at this location and the posterior parietal peritoneum is adjacent to the abdominal wall.

It may be difficult, in the absence of ascites, to differentiate parietal peritoneal deposits from their visceral counterparts at locations where the two leaves are adjacent (e.g., a deposit within the parietal peritoneum covering the lateral abdominal wall or within the visceral peritoneum covering the liver). For the sake of simplicity, some examples of parietal peritoneal deposits are shown; the rest are included within their peritoneal space (Figure 4).

If the deposit lies within a fat-containing peritoneal space, the spectrum of presentation ranges from nodular focal fat stranding to irregular haziness, evolving towards solid lesions. These solid lesions may initially be millimetric, appearing as either solitary or multiple soft-tissue nodules that eventually grow and merge to form plaques or sheets, and then masses. Most commonly, PC appears as a combination of all these findings.

Peritoneal spaces are classified as supra- or inframesocolic [29,30]. The anatomic landmark that enables this division is the mesentery of the transverse colon (transverse colon mesocolon). Unfortunately, this is also an easy route for carcinomatosis spread, as it communicates on both sides with other ligaments and centrally with the small bowel mesentery.

**Figure 5:**
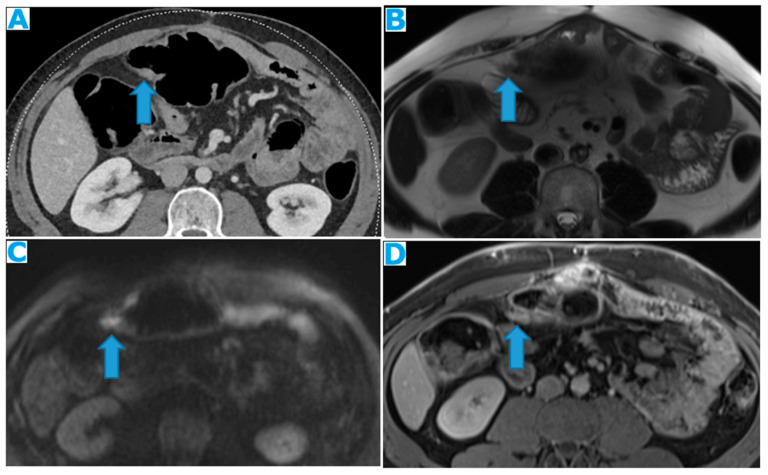
Axial CE-CT (**A**), axial T2WI (**B**), DWI (**C**), axial CE portal phase FST1WI (**D**). PC from gastric adenocarcinoma: subtle deposit within the transverse mesocolon (arrow) using CT; more conspicuous using MR.

Deposits within the transverse mesocolon appear either on its mesentery (Figure 5) or/and on the serosa (visceral peritoneum) covering the transverse colon [30]. Differentiation between them may not always be feasible. Deposits may cause different degrees of luminal stenosis with or without signs of bowel obstruction.

**Figure 6:**
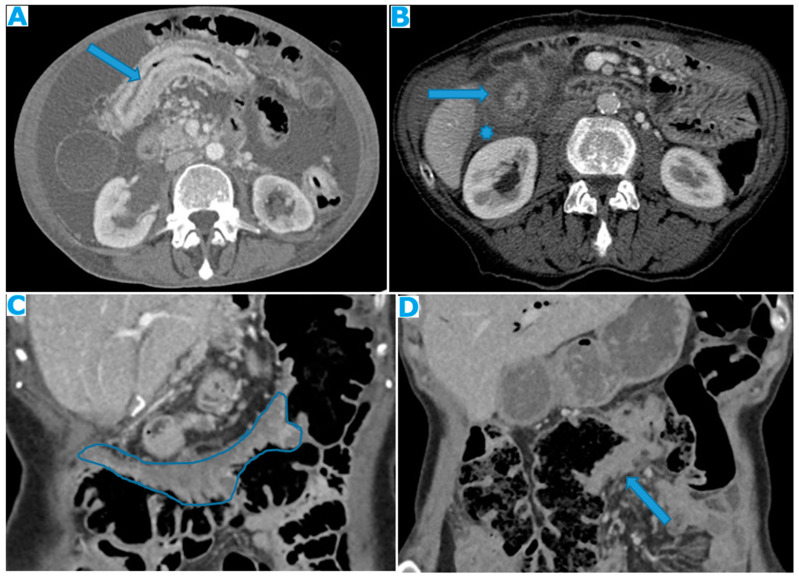
Axial CE-CT (**A**)**.** PC from breast carcinoma: deposits within the transverse colon serosa (arrows), causing luminal stenosis. Axial CE-CT (**B**). PC from breast carcinoma: layered deposits within the transverse colon serosa (arrow); notice the concentric pattern and the luminal stenosis. Ascites (*). Coronal CE-CT MPR (**C**,**D**). (**C**): PC from signet ring cell gastric carcinoma. Diffuse serosal infiltration of the transverse colon. (**D**): PC from breast carcinoma: segmental parietal thickening of the transverse colon due to nodular deposits within the serosa (arrow). Observe how layered deposits in (**C**) blend in with the bowel contour and may be more difficult to detect than nodular deposits in (**D**).

Imaging features of serosal bowel deposits include nodular lesions, segmental parietal thickening, and diffuse infiltration. Their detection may be an arduous task, more so if bowel is not sufficiently distended. In addition, it is important to note that layered implants blend in with the bowel contour, whereas nodular deposits alter it and are thus easier to detect (Figure 6).

This description of serosal bowel deposits has the same validity for the rest of the gastrointestinal tract.

Next, we use a cranial-to-caudal and lateral-to-medial approach to review the peritoneal spaces.

### 3.1. Supramesocolic Spaces

Supramesocolic cavity comprehends the subphrenic, perihepatic, and perisplenic spaces; periportal space, lesser omentum, lesser sac, and right subhepatic space. These locations need to be carefully assessed using multiplanar reconstructions, as deposits within them may require complex surgery or, in some cases, may be considered unresectable.

#### 3.1.1. Subphrenic Spaces

**Figure 7:**
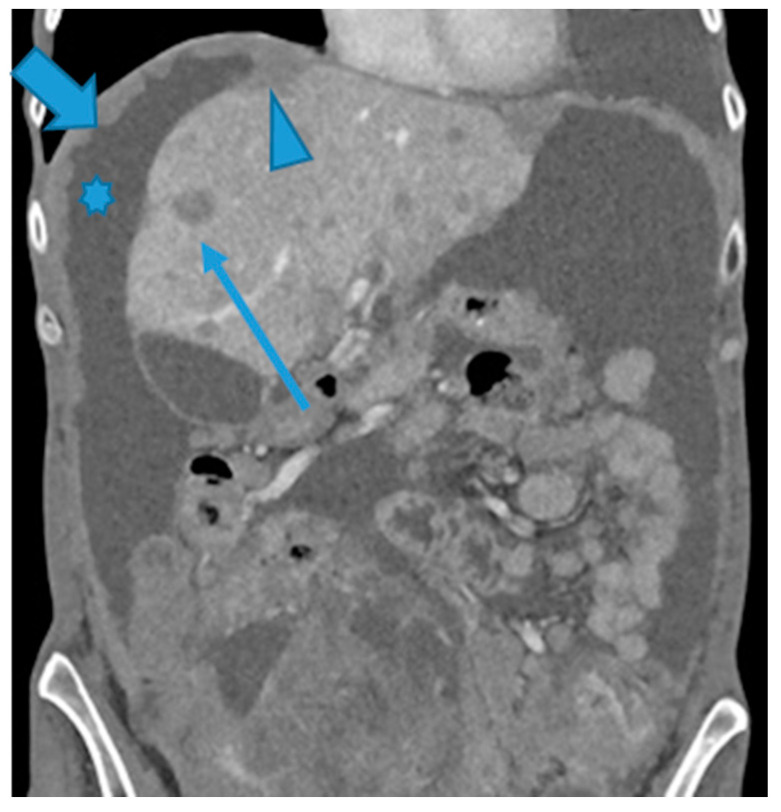
CE-CT coronal MPR. PC from ovarian carcinoma: multiple bilateral diaphragmatic nodular deposits (arrows). Notice how useful ascites (*) is when distinguishing peritoneal deposits within the parietal peritoneum from deposits within the visceral (hepatic) peritoneum (arrowhead). Hepatic metastases (thin arrow).

**Figure 8:**
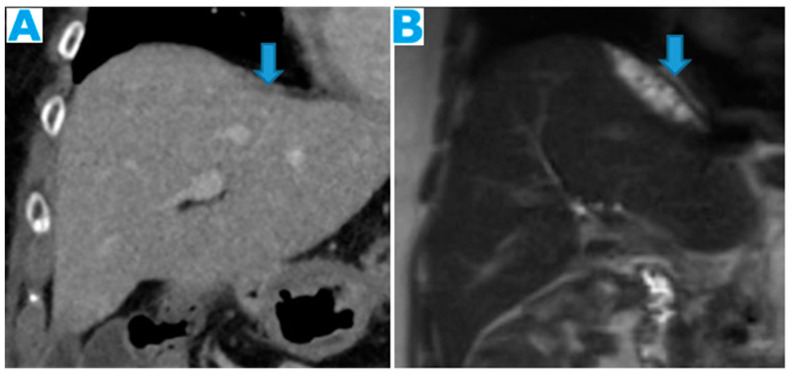
Coronal T2WI ((**A**), from five years prior) and CE-CT coronal MPR ((**B**), current follow-up). PC from ovarian granulosa cell tumour: Notice, in (**B**), a deposit within the right subphrenic space, bulging into the hepatic capsule (arrow). See how subtle it originally was in (**A**) (arrow); it could easily be overlooked upon axial imaging.

Imaging features include the thickening and pathological enhancement of the diaphragm, nodules, and masses (Figure 7 and Figure 8).

#### 3.1.2. Perihepatic and Perisplenic Spaces

Deposits can be identified as a continuum from abnormal peritoneal enhancement to subtle nodularity and well-defined nodules, often showing a biconvex morphology.

Perihepatic deposits may occur on the superficial visceral peritoneum that surrounds most of the liver surface (except for the liver bare area, the porta hepatis, and the attachment site of the gallbladder to the liver) and/or underneath the Glisson capsule, within the subcapsular space. Glisson’s capsule is a thick fibrous membrane that lies deep in the visceral peritoneum. It is assumed that deposits infiltrate the liver capsule upon deposition on the visceral peritoneum. The liver capsule covers the entire hepatic surface, including the periportal space, and is in communication with the lesser omentum, thus becoming another route for deposits to reach the subcapsular space. Both the periportal space and the lesser omentum are reviewed next.

**Figure 9:**
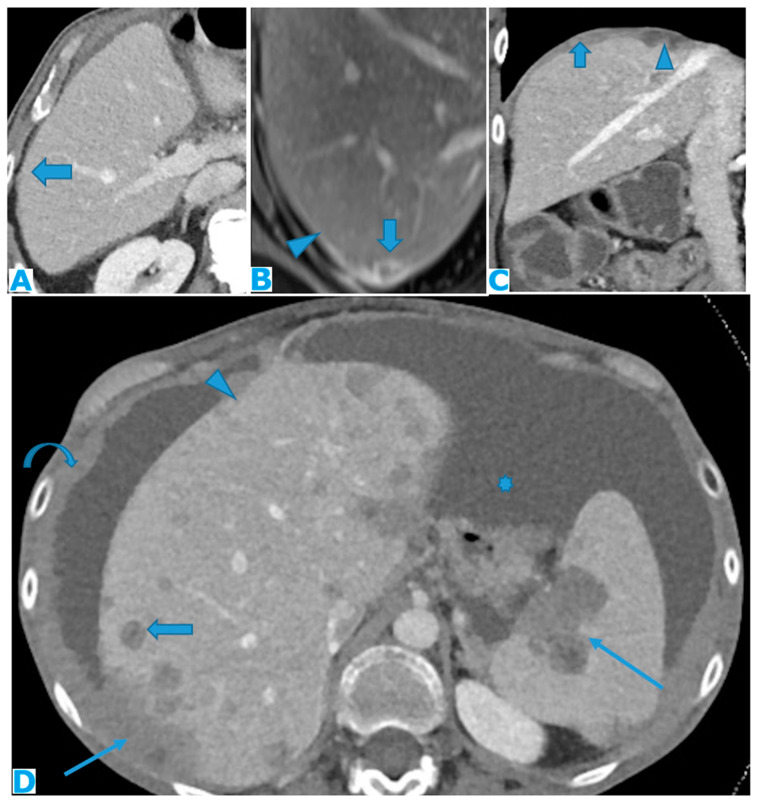
Axial CE-CT (**A**): PC from colon carcinoma: note the subtle irregularities in the liver contour caused by the deposit seeding within the peritoneum covering the hepatic surface. CE portal phase FST1WI (**B**): PC from cervical carcinoma: notice the difference between the linear subcapsular deposits (arrowhead) and the biconvex subcapsular deposit with parenchymal invasion (arrow): observe the scalloped appearance of the underlying parenchyma. CE-CT coronal MPR (**C**): PC from colon carcinoma: thickening of the right diaphragm caused by deposit seeding (arrow). Notice the difference with the subcapsular deposits that scallop the liver contour (arrowhead). Axial CE-CT (**D**): PC from ovarian carcinoma: subcapsular deposits with parenchymal invasion (thin arrows), both hepatic and splenic. Observe how they differ from hepatic metastases, which are well defined and completely surrounded by parenchyma (arrow). Note also the perihepatic (arrowhead) and right subphrenic deposits (curved arrow). Ascites (*).

**Figure 10:**
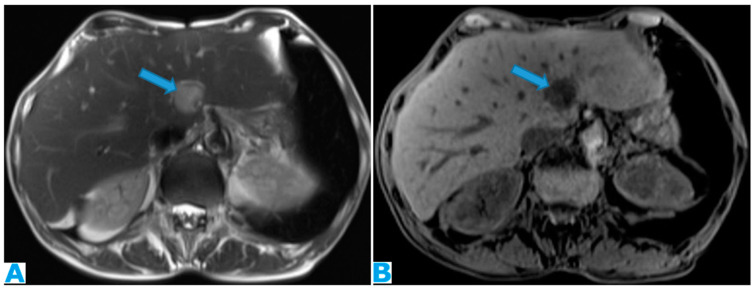
Axial T2WI (**A**); axial NE FST1WI (**B**). PC from ovarian serous carcinoma: subcapsular hepatic deposit, presenting a fatty surrounding plane (arrow) that excludes secondary hepatic invasion.

In the event of subcapsular deposits, secondary parenchymal invasion may occur, resulting in a characteristic scalloping of the underlying parenchyma (Figure 9). Despite this sign, it may be difficult to distinguish between solely subcapsular deposits and those with parenchymal invasion. A sign that has been found to be highly sensitive to rule out secondary hepatic invasion is the presence of a well-defined interface between the lesion and the liver and/or a clear plane, either fatty or from ascites [31] (Figure 10).

#### 3.1.3. Periportal Space

**Figure 11:**
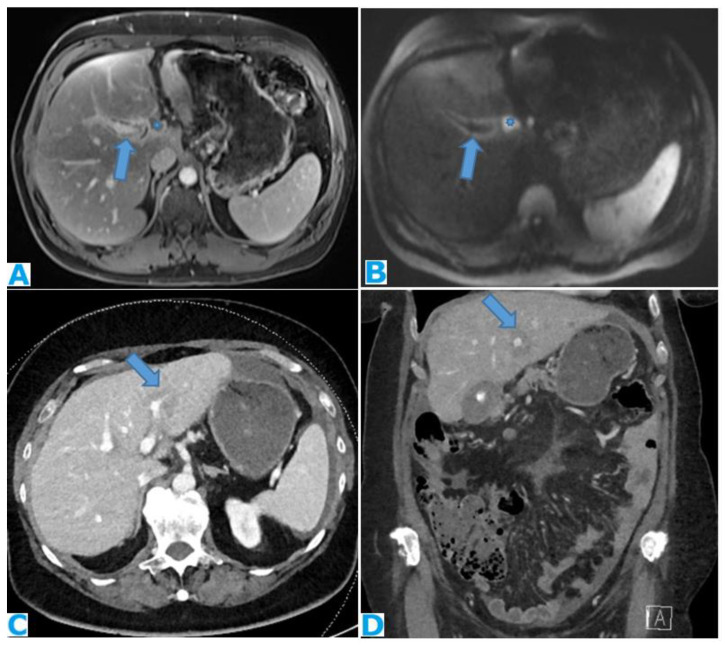
DWI (**A**); axial CE portal phase FST1WI (**B**). PC from colon carcinoma: deposits within the periportal space. Observe the diffusion restriction and enhancement around the periportal space (arrows); also note the nodular deposit (*). Axial CE-CT (**C**) and coronal MPR (**D**). PC from ovarian carcinoma: note the periportal deposit as a soft tissue mass around the left portal branch, which is more conspicuous in the MPR.

Periportal deposits need to be differentiated from parenchymal metastases. Deposits within the periportal space appear as nodular or plaque-like lesions, predominantly at the porta hepatis and along the left branch of the portal vein; they are usually ill defined and not circumferentially surrounded by hepatic parenchyma, unlike their intraparenchymal counterparts (Figure 11).

**Figure 12:**
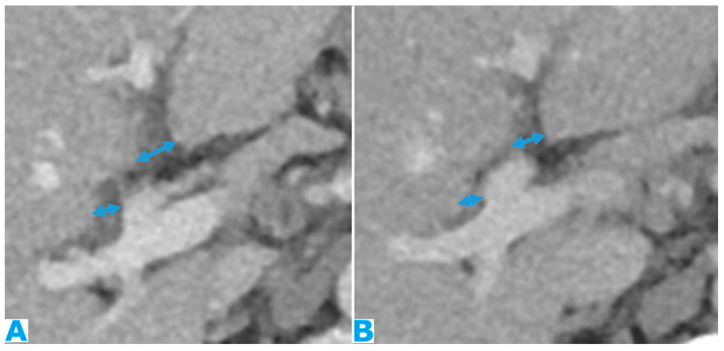
Axial CE-CT ((**A**), current study, and (**B**), from one year prior). PC from gastric adenocarcinoma: subtle soft tissue mass occupying the periportal space. Observe the periportal space enlargement (arrows), which is more conspicuous if compared to the previous CT.

A proven useful tip is the distention of the periportal space over time due to the presence of deposits. As with any radiological examination, comparison with prior images in the setting of peritoneal carcinomatosis is mandatory (Figure 12).

**Figure 13:**
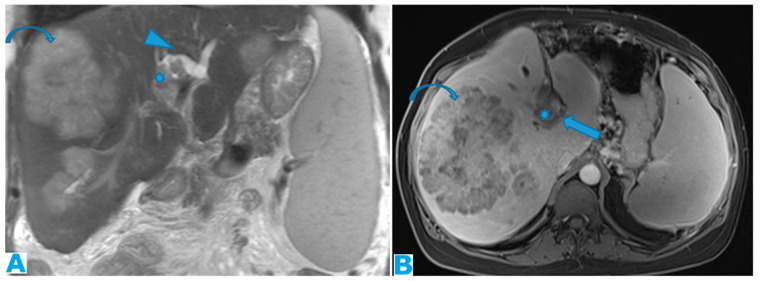
Coronal T2WI (**A**); axial CE portal phase FST1WI (**B**). PC from colon adenocarcinoma: periportal deposit (*) that causes segmental intrahepatic biliary dilatation (arrowhead) and portal vein compression (arrow). The patient had a known portal hypertension due to massive hepatic metastases (curved arrow) and splenomegaly.

Deposits within this location may cause biliary obstruction, which usually presents at a late stage. Dilatation of the intrahepatic biliary ducts (usually segmentary) with no identifiable cause should raise the possibility of deposits within the periportal spaces. Another secondary finding may be a progressive compressive effect on the portal vein over time (Figure 13).

#### 3.1.4. Lesser Omentum

This portion of the peritoneum suspends the liver and the lesser curvature of the stomach and separates the first two centimetres of the duodenum from the liver. It is formed by the gastrohepatic and hepatoduodenal ligaments.

**Figure 14:**
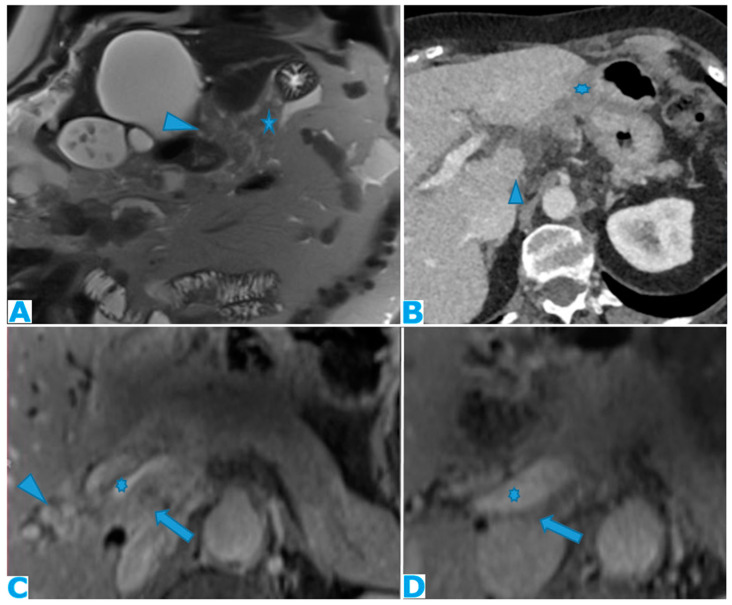
Axial CE-CT (**A**). PC from ovarian carcinoma: deposits within the gastrohepatic (*) and hepatoduodenal ligaments (arrowheads). Coronal T2WI (**B**). PC from ovarian carcinoma: deposits within the lesser omentum: its two components are identified—the gastrohepatic (*) and the hepatoduodenal (arrowhead) ligaments. Axial CE portal phase FST1W1 (**C**,**D**): the current study (**C**) and from one year prior (**D**), for comparison. PC from colon adenocarcinoma: deposits within the gastrohepatic ligament (lesser omentum) (arrows). Note the compression of the portal vein (*) in (**C**) and the development of collateral vessels (arrowheads).

Deposits within this space show features that are common to any fat-containing space. Furthermore, since the porta hepatis runs in the hepatoduodenal ligament, biliary obstruction or portal vein compression may be found to be indirect signs of PC (Figure 14).

As previously seen, once the disease is in the lesser omentum it can easily spread to the periportal space thanks to the surrounding connective tissue of the Glisson sheath, which makes them continuous. This is a particularly important route for the spread of pancreatic and gastrointestinal tumours.

#### 3.1.5. Lesser Sac

**Figure 15:**
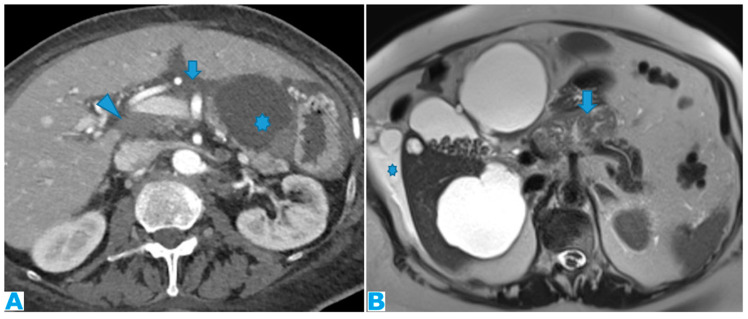
Axial CE-CT (**A**). PC from ovarian carcinoma: mass-like deposit within the lesser sac (*). Also, note the seeding within the lesser omentum (arrow). Portacaval lymph node (arrowhead). Axial T2WI (**B**). PC from ovarian carcinoma: mass-like deposit within the lesser sac (arrow). Ascites (*).

Potential space between the pancreas and the stomach. Its distention, either by a solid lesion or by ascites, as is reviewed later, is a sign of its involvement by PC (Figure 15). It communicates with the rest of the peritoneal cavity (greater sac) through an opening immediately posterior to the lesser omentum, the foramen of Winslow.

#### 3.1.6. Right Subhepatic Space

**Figure 16:**
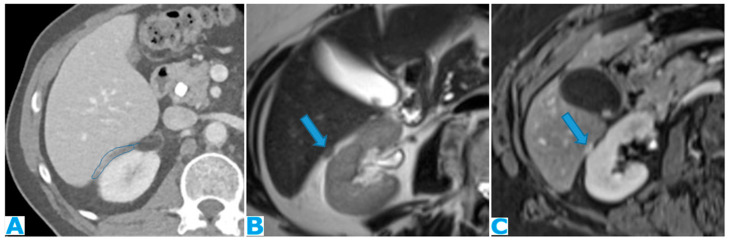
Axial CE-CT (**A**): PC from breast carcinoma: deposits within the subhepatic space as reticulation of its fatty content. Axial T2WI CE-CT (**B**); CE portal phase FST1WI (**C**): PC from colon carcinoma: nodular deposit within the subhepatic space (arrow).

Pouch inferior to segment VI of the liver. It communicates with the right subphrenic space, the right paracolic gutter and the lesser sac. Deposits within this space tend to be more subtle, partly due to small size of this space, and range from an ill-defined outer hepatic contour to focal fat stranding and nodules (Figure 16).

### 3.2. Inframesocolic Spaces

Inframesocolic spaces are also described using the same cranial-to-caudal and lateral-to-medial approach: greater omentum, paracolic gutters, small bowel mesentery, sigmoid mesocolon, and pelvis recesses.

#### 3.2.1. Greater Omentum

The greater omentum is the main peritoneal fold. It connects the stomach to the anterior surface of the transverse colon and then extends caudally into the pelvis covering the small bowel loops. It lies mainly inferior to the transverse colon mesocolon, although its smaller cranial portion (the gastrocolic ligament) is within the supramesocolic space.

**Figure 17:**
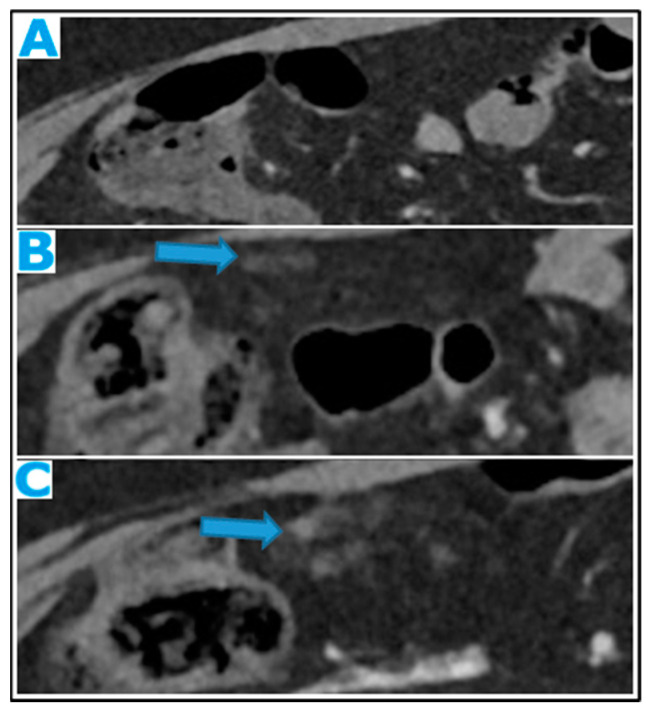
Axial CE-CT (**A**) from one year prior (disease-free peritoneum); (**B**) from four months prior; (**C**) current follow-up. Note the early stages and evolution of omental deposits (arrows).

**Figure 18:**
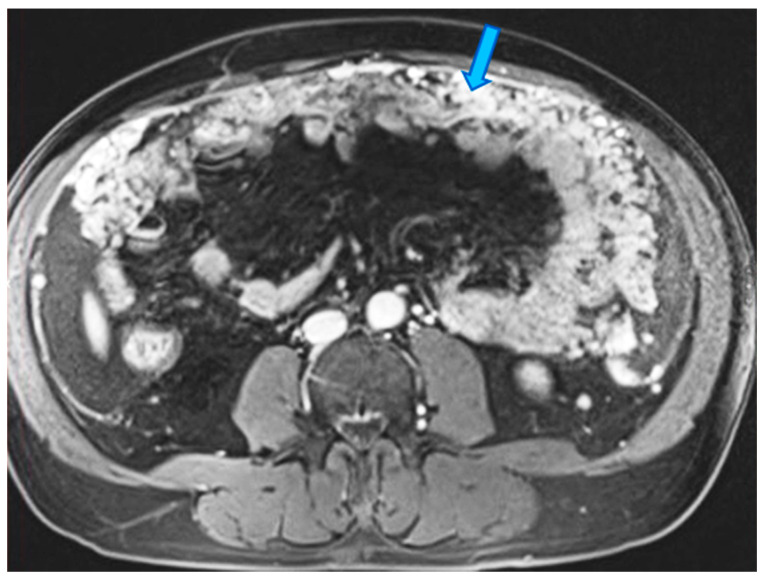
Axial CE portal phase FST1W. PC from melanoma: omental cake (arrow) replacing the omental fat.

The uniqueness of the imaging features of deposits in this location is the omental cake, which occurs when nodular deposits collide (Figure 17) and blend with one another, boosting a fibrotic response and replacing the omental fat (Figure 18).

**Figure 19:**
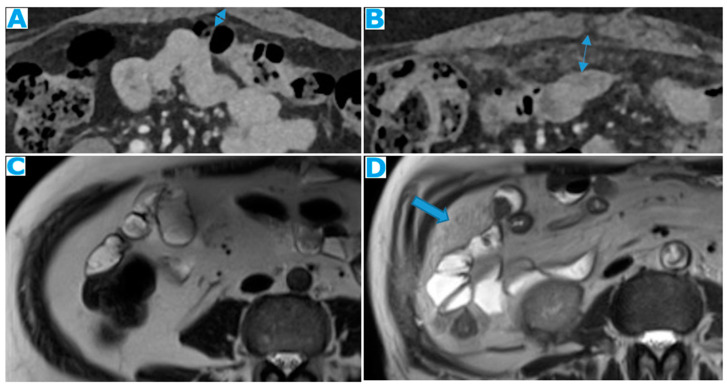
Axial CE-CT ((**A**) from one-year prior; (**B**) current follow-up). PC from breast carcinoma: note the omental infiltration on (**B**) associated with a posterior displacement of the small bowel loops (arrows). Axial T2WI ((**C**) peritoneal disease-free study from two years prior; (**D**) current follow-up). PC from renal cell carcinoma: enlargement of the fatty content and mass effect due to omental seeding (arrow) that may be more conspicuous than the actual deposits.

A helpful diagnostic sign as the infiltration progresses is the subsequent displacement of the bowel loops. Enlargement of the fatty content and mass effect due to omental seeding may be more apparent than the actual deposits (Figure 19).

**Figure 20:**
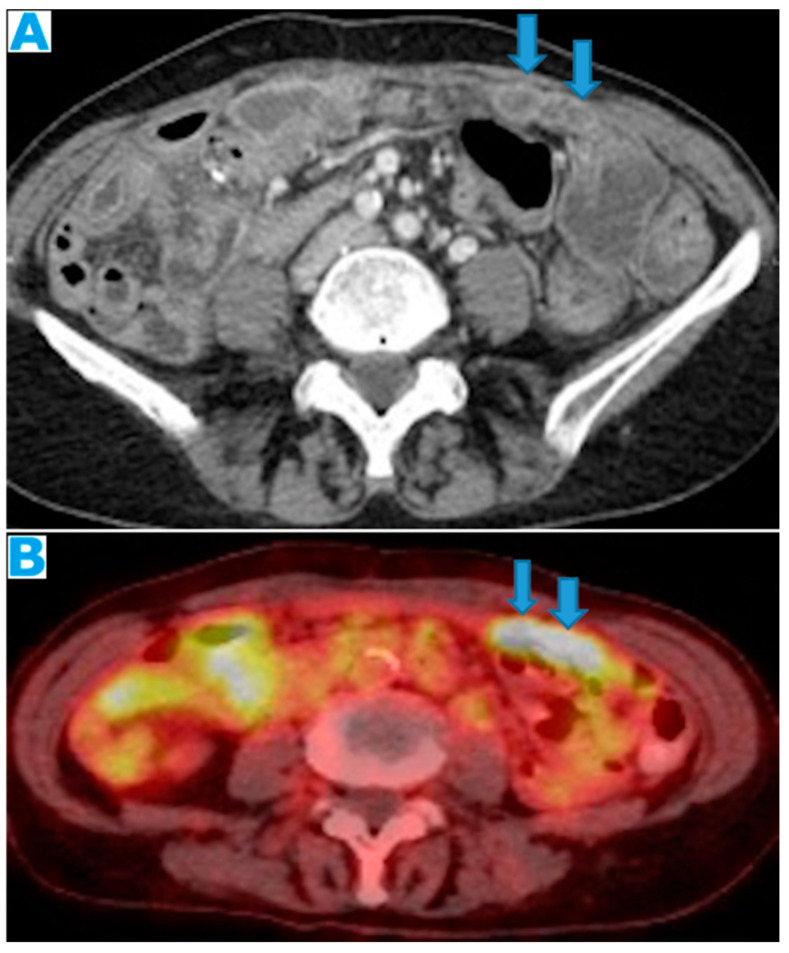
Axial CE-CT (**A**); FDG-PET-CT (**B**). PC from colon adenocarcinoma: nodular deposits within the omentum (arrows) that were originally mistaken for SB loops upon CT due to the scarce intrabdominal fat.

Omental deposits are more easily detected using MR. However, in the early stages and especially in thin patients, CT appears to perform better, although the scarce fatty content in thin patients may negatively contribute to the identification of peritoneal deposits (Figure 20).

#### 3.2.2. Small Bowel Mesentery

The small-bowel mesentery is a fan-shaped peritoneal double layer that surrounds the small bowel and then extends diagonally from the ligament of Treitz in the left upper quadrant to the ileocecal junction, anchoring at these two locations and at the posterior parietal peritoneum.

**Figure 21:**
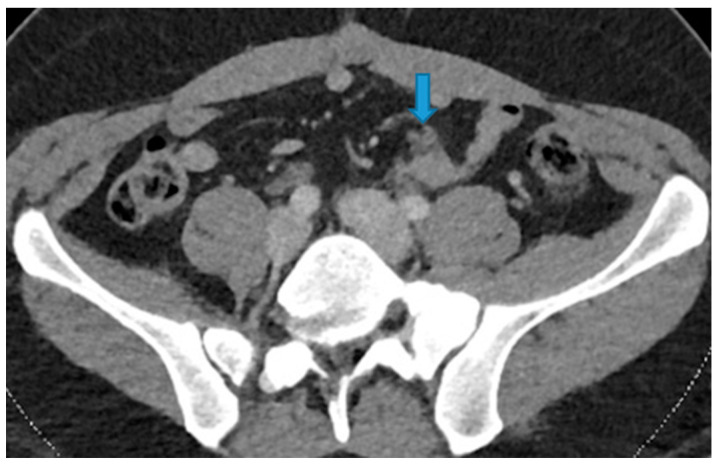
Axial CE-CT. PC from colon adenocarcinoma: nodular deposit within the mesentery (arrow).

**Figure 22:**
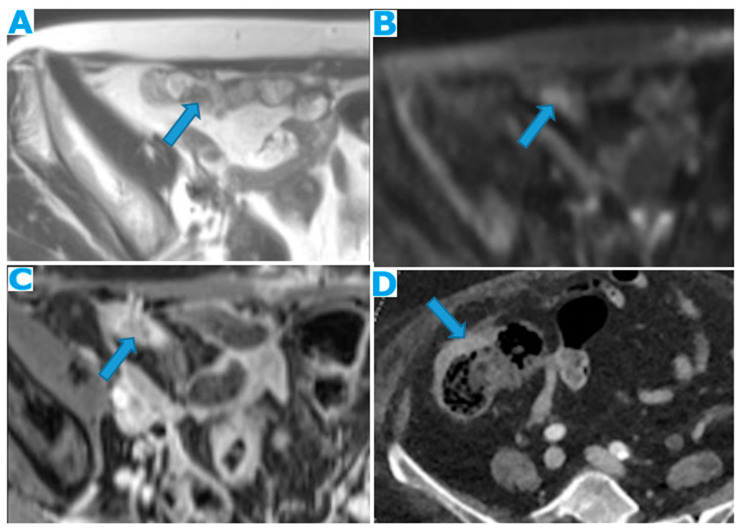
Axial T2WI (**A**); axial DWI (**B**); CE portal phase FST1WI (**C**). PC from duodenal adenocarcinoma: deposits within the distal ileum serosa (arrow). Axial CE-CT (**D**). PC from breast carcinoma: deposits within the caecal serosa (arrow).

**Figure 23:**
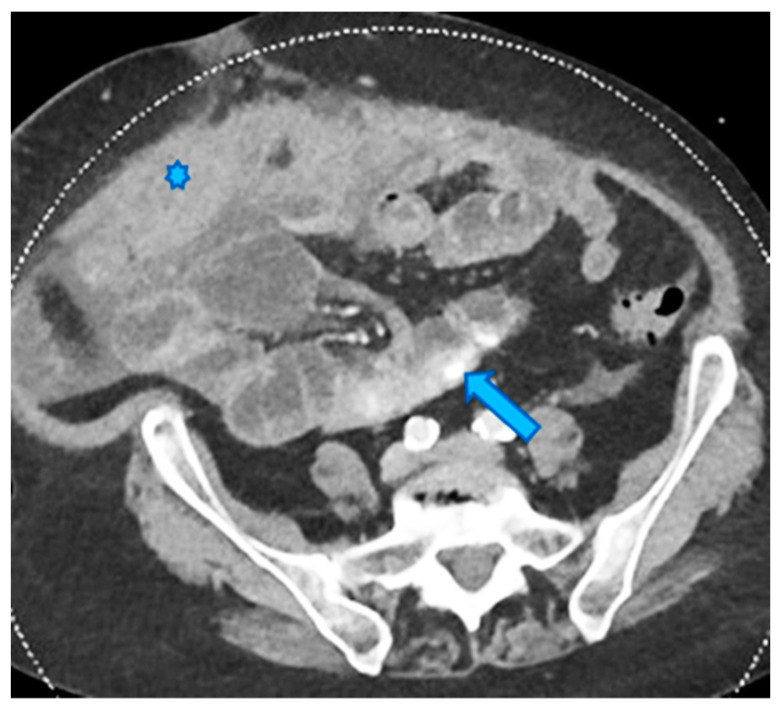
Axial CE-CT. PC from ovarian carcinoma: mesenteric seeding. Mesenteric involvement may occur as a combination of deposits involving both the mesentery and the bowel serosa, as in this case. Observe the clustered SB loops’ appearance. The calcified content of some of the deposits enhances their presence (arrow). Omental deposits (*).

**Figure 24:**
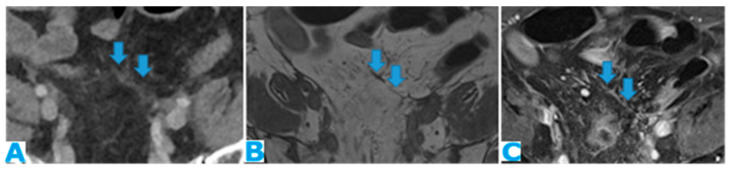
Axial CE-CT (**A**); axial T2WI (**B**); axial CE portal phase FS T1WI (**C**). PC from endometrial carcinoma: deposits seeding within the mesenteric leaves (arrows).

**Figure 25:**
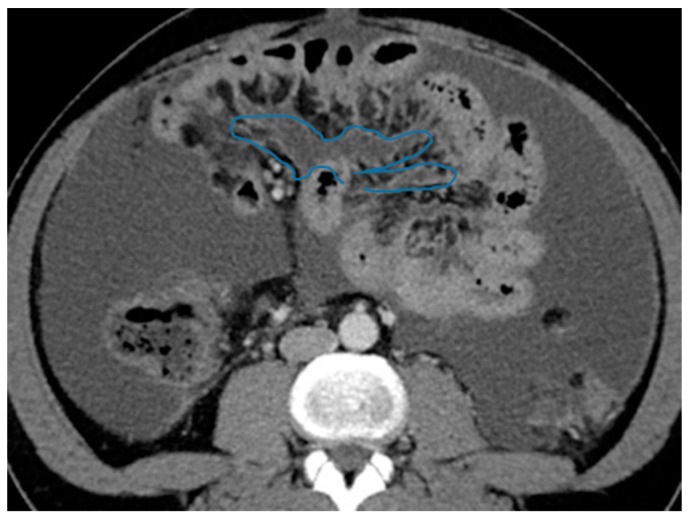
Axial CE-CT. PC from colon adenocarcinoma: involvement of the mesenteric leaves (note the nodular thickening and enhancement, highlighted in blue) that becomes more apparent with ascites.

**Figure 26:**
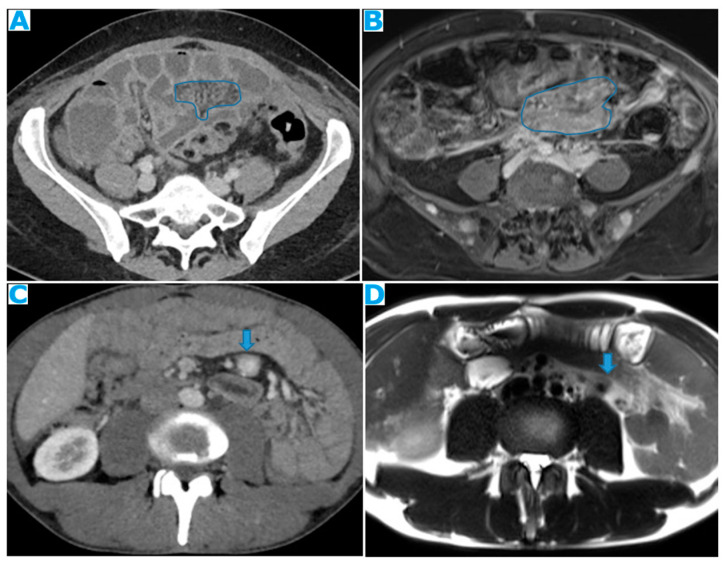
Axial CE-CT (**A**). PC from stomach adenocarcinoma: stellate mesentery. Axial CE portal phase FST1WI (**B**). PC from lobular breast adenocarcinoma: stellate mesentery; note the perivascular distribution. Axial CE-CT (**C**); axial T2WI (**D**). PC from stomach adenocarcinoma: isolated perivascular deposit within the mesentery as a soft-tissue mass surrounding a branch of the SMV (arrow).

Mesenteric deposits show a rather unique and more complex appearance when compared to deposits elsewhere in the abdominal cavity. Deposits may be found in the following locations:

- Within the mesenteric fat: as in the rest of the fat-containing peritoneal spaces, they may range from focal nodular fat stranding to irregular haziness to nodules and masses (Figure 21).

- Within the SB and caecal serosa: deposits may also lie within the serosa covering the small bowel and the caecum (Figure 22).

- Involving both the mesentery and the serosa: as in the transverse mesocolon, deposits may appear both within the mesentery and the serosa covering the small bowel loops and the caecum (Figure 23).

- Within the mesenteric leaves: Deposits within the mesenteric leaves may go unperceived. The nodular thickening and enhancement of the mesenteric leaves is usually more conspicuous using MR but can be also spotted using CT and becomes more noticeable when accompanied by ascites (Figure 24 and Figure 25).

- Stellate mesentery: Diffuse mesenteric infiltration leads to a stellate appearance, which is commonly associated with breast (especially lobular carcinoma) [32], gastric, pancreatic, and ovarian tumours [33]. This deposition pattern follows the distribution of the mesenteric vessels, causing the thickening and rigidity of perivascular bundles (Figure 26).

**Figure 27:**
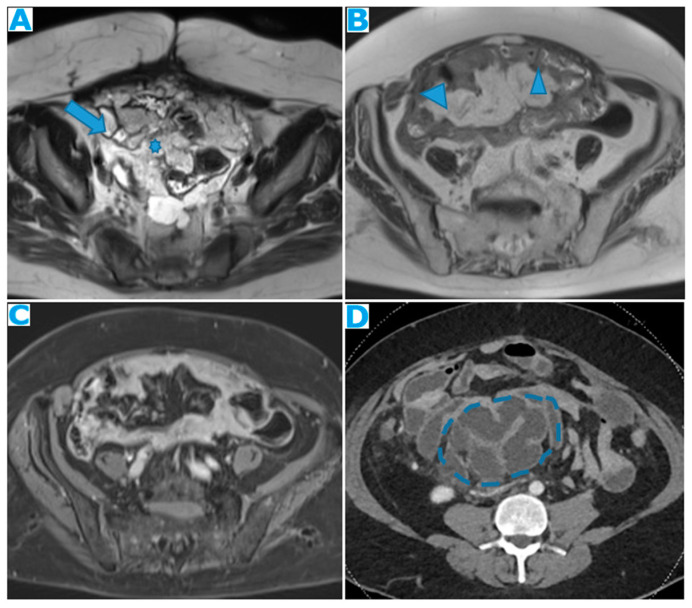
Axial T2WI (**A**). PC from mucinous adenocarcinoma of the urachus: notice the hyperintense mesenteric deposits (*) (signal due to mucin content) and how the SB loops appear separated and angulated (arrow). Axial T2WI (**B**); axial CE portal phase FST1WI (**C**). PC from breast carcinoma: mesenteric deposits (arrowheads on (**B**)) make SB loops appear thickened and separated. Notice how deposits are more conspicuous in T2WI due to its high tissue contrast. Axial CE-CT (**D**). PC from ovarian carcinoma: clustered small bowel loops as the end point of mesenteric seeding.

**Figure 28:**
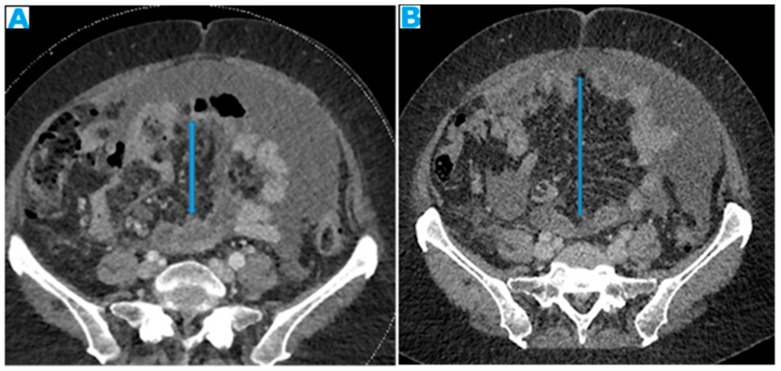
Axial CE-CT: current study (**A**) and from six months prior (**B**). PC from colon carcinoma: the mesentery becomes fibrotic as the seeding evolves and, secondary to the retraction effect, a decrease in the size of the mesenteric fat occurs (arrows). This effect may be more conspicuous than the actual deposits.

As a result of tumour infiltration, the mesentery becomes rigid and loses its usual free wavering. SB loops appear thickened, with restricted distensibility, looking initially separated and angulated and, finally, clustered (Figure 27). Over time, the retraction caused by the deposits causes a decrease in the size of the mesenteric fat (Figure 28). These effects on the SB loops and the mesenteric fat may be more conspicuous than the actual deposits.

**Figure 29:**
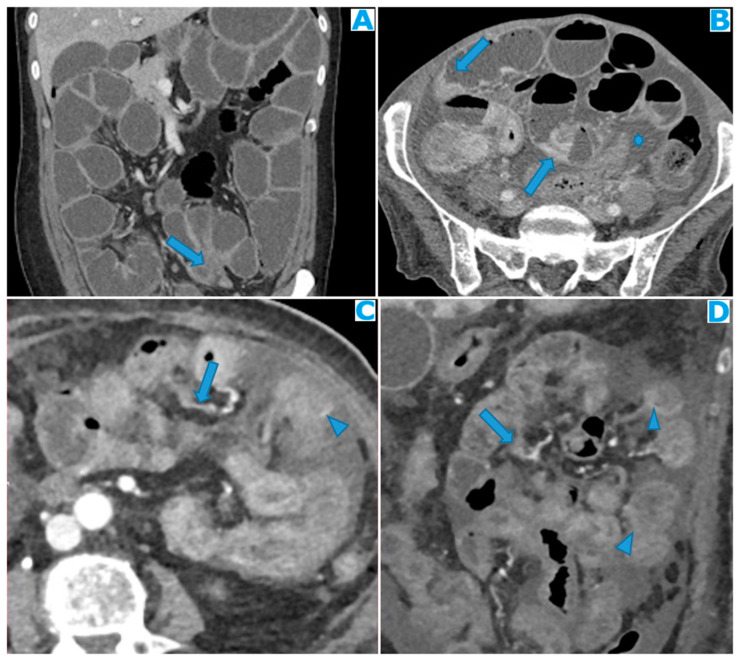
Coronal MPR (**A**). PC from mucinous colon adenocarcinoma: solitary mesenteric deposit (arrow) as the cause of an SB obstruction, involving several loops. Axial CE-CT (**B**). PC from breast carcinoma: SB multifocal obstruction due to several deposits within the SB serosa (arrows). Ascites (*). Axial CE-CT (**C**) and coronal MPR (**D**). PC from adenocarcinoma of the urachus: severe mesenteric infiltration causing SB ischemia as distal SMA and SMV branches are compressed and infiltrated by deposits (arrows). Observe the SB loops thickening and the heterogenous and patchy bowel wall enhancement (arrowheads) due to the ischemia.

The main complication of deposits lying within this space is bowel obstruction, which usually occurs at a late stage, and its diagnosis is not challenging. However, it may also be the presenting sign. It typically involves more than one small bowel loop, and the degree of occlusion may vary. Another, less frequent, complication is bowel ischaemia due to perivascular infiltration (Figure 29).

#### 3.2.3. Paracolic Gutters

Peritoneal recesses occur between the colon, partially covered by the posterior parietal peritoneum (on its anterior, medial, and lateral walls) and the lateral parietal peritoneum on each side. They constitute the attachments of the ascending and descending colon to the posterior parietal peritoneum.

Both are in continuity with the peritoneal spaces in the pelvis. Superiorly, the right paracolic gutter communicates with the right subphrenic and right subhepatic spaces, whereas the left paracolic gutter, which is much smaller, is partially separated from the left subphrenic space by the phrenicocolic ligament (Figure 1b).

**Figure 30:**
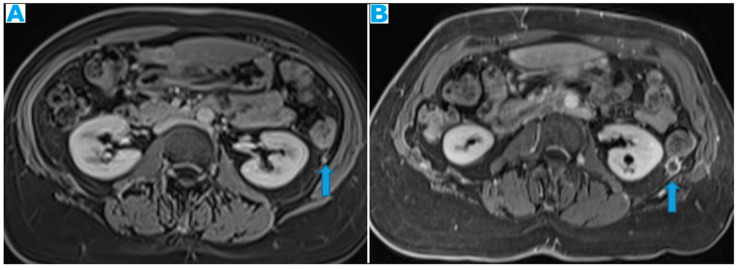
CE portal phase FST1WI ((**A**), from four months prior; (**B**), current study). PC from colon adenocarcinoma: Nodular deposit within the left paracolic gutter that was undercalled (arrow). See the growth in (**B**) (arrow).

The infiltrated peritoneum typically appears to be nodularly thickened, showing pathological enhancement (Figure 30).

**Figure 31:**
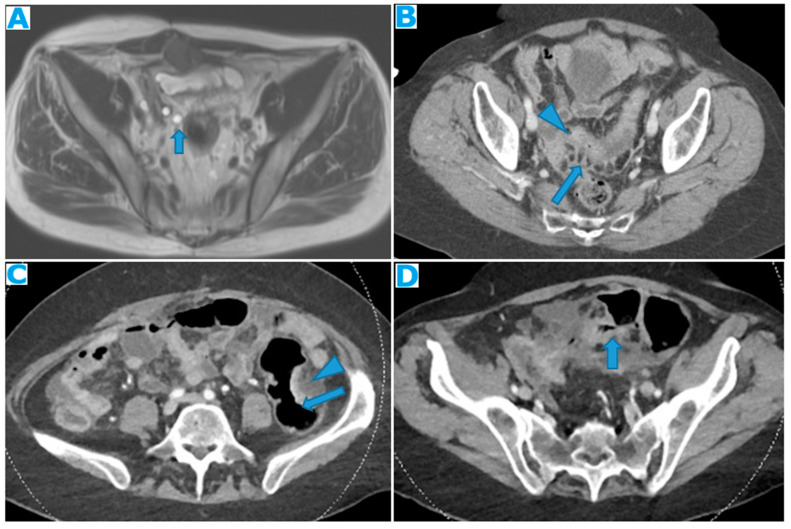
Axial T2WI (**A**). PC from mucinous adenocarcinoma of the terminal ileum: hyperintense nodular deposits (due to mucin content) within the sigmoid mesocolon. Axial CE-CT (**B**). PC from undifferentiated carcinoma of unknown origin: deposits within both the sigmoid mesocolon (arrows), outlining the epiploic appendices, and within the sigmoid serosa, making the sigmoid colon appear thickened (arrowheads). Axial CE-CT (**C**,**D**). PC from colon adenocarcinoma: (**A**). Observe in (**C**) the seeding involving the sigmoid mesocolon (arrowhead) and the serous layer of the bowel (arrow), partially obstructing the sigmoid lumen. In (**B**), the two components cannot be differentiated but luminal stenosis is clearly identified (arrow).

The oblique orientation of the small bowel mesentery described earlier divides the inframesocolic compartment into two compartments, right and left, with the latter being larger. The only structure standing in the way of free communication between the left inframesocolic space and the pelvis is the sigmoid mesocolon, thus the reason why it constitutes a common site of deposits, as it is an area of arrested flow. Deposits within the sigmoid mesocolon lie on its fat and/or on the serosa, and differentiation between them may not always be feasible. Sigmoid luminal stenosis with/without signs of obstruction is a frequent consequence of the seeding (Figure 31).

The mesentery of the appendix is anchored to the lower end of the small bowel mesentery, close to the ileocecal junction and the tip of the appendix. Despite its small size, it may be an important site in appendiceal neoplasms: in case of rupture, deposits will likely appear there first.

#### 3.2.4. Peritoneal Recesses of the Pelvis–Ovarian Metastases

Deposits within the pelvis may be quite tricky to find, as the anatomy is rather complex and there are several structures that fit in a small cavity; therefore, careful exploration using multiplanar reconstructions is recommended.

**Figure 32:**
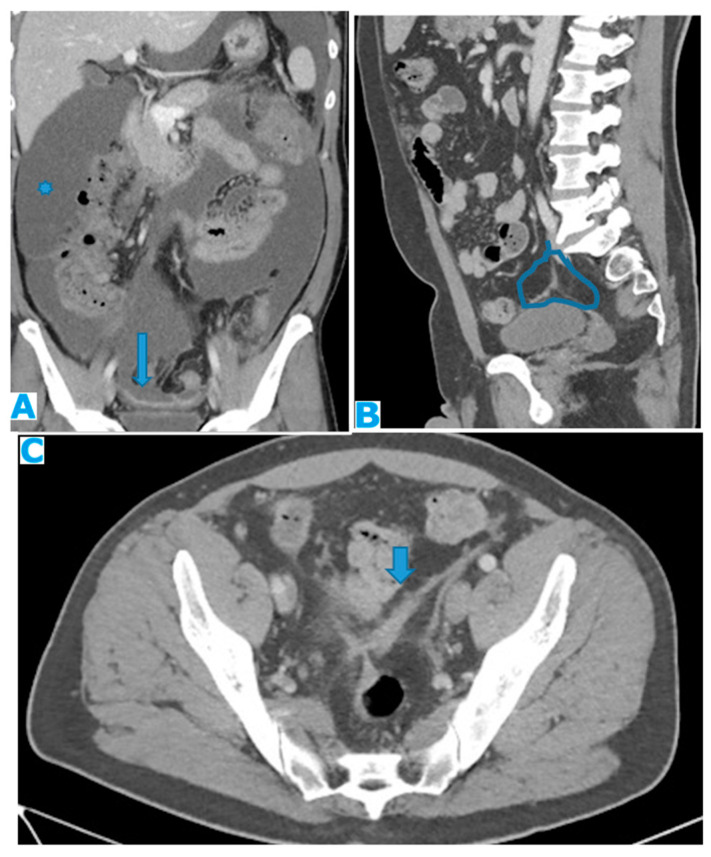
CE-CT coronal MPR (**A**). PC from colon carcinoma: observe how the parietal peritoneum does not reach the pelvis floor. Ascites (*). Deposits within the pelvic reflexion (arrow). Sagittal MPR (**B**); axial CE-CT (**C**). PC from adenocarcinoma of the appendix: irregularly thickened peritoneal reflexion due to seeding. Observe in the sagittal MPR how the peritoneal reflexion covers the dome of the urinary bladder and then descends, following its posterior wall.

The parietal peritoneum that covers the abdominal wall goes down to the pelvis, where it does not reach the pelvis floor, as it reflects on the pelvis organs (peritoneal reflexion). The peritoneal reflexion covers the dome of the urinary bladder, then descends along its posterior wall and laterally forms paravesical spaces, a fold over the ureters; in men, it also covers the deferent ducts and the seminal vesicles. It continues towards the rectum and then ascends, partially cloaking the upper and middle rectum (thus subperitoneal; the rest is extraperitoneal) and the lateral pelvic walls (Figure 32). In women, it also coats the uterine fundus and body and the posterior part of the vagina and extends laterally (broad ligament), wrapping up the tubes and suspending the ovaries.

Two blind-end pouches are found in women—the uterovesical, anteriorly, and the rectouterine, posteriorly—while there is only one in men, the rectovesical [7].

**Figure 33:**
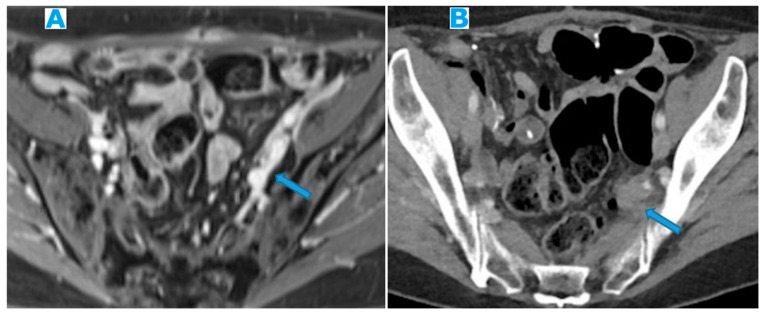
Axial CE portal phase FST1WI (**A**). PC from duodenal adenocarcinoma: deposits seeding within the peritoneum that covers the left pelvic wall, which looks diffusely thickened (arrow). Axial CE-CT (**B**). PC from undifferentiated caecal adenocarcinoma: nodular deposits within the peritoneum that covers the left pelvic wall (arrow).

**Figure 34:**
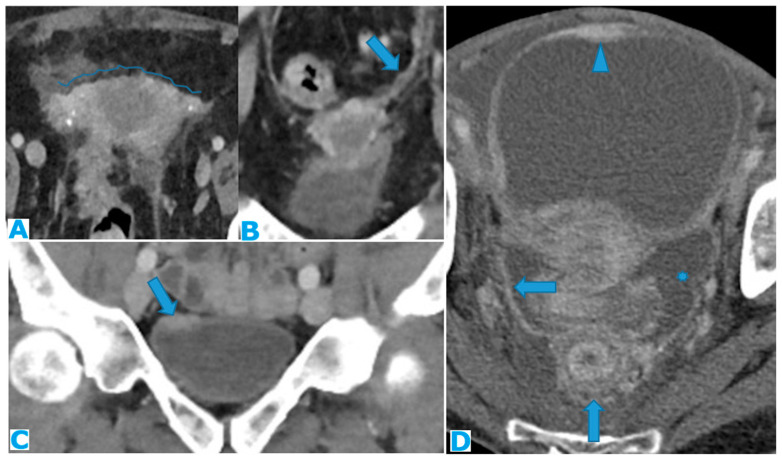
Axial CE-CT (**A**); CE-CT coronal MPR (**B**). PC from breast carcinoma: the peritoneal reflexion covers the uterine fundus and body and the posterior part of the vagina and extends laterally (broad ligament, arrow in (**B**)). Note the nodular appearance of the peritonealised uterine surface and the broad ligaments (arrow in (**B**)) due to deposit seeding. CE-CT coronal MPR (**C**). PC from cardia adenocarcinoma: deposits within the peritoneal reflexion covering the vesical dome (arrow). Axial CE-CT (**D**). PC from breast carcinoma: notice the peritoneal seeding on the peritonealised surfaces of the bladder (arrowhead), rectum (vertical arrow) and within the peritoneum that covers the pelvic walls (horizontal arrow). Ascites (*).

Pelvic organs, except for the tubes, which are intraperitoneal, are only superiorly and laterally covered by the peritoneum. Thus, deposits can be identified as enhancing nodular peritoneal thickenings on the pelvic walls (Figure 33) or within the peritoneal reflexion, either surrounded by fat or on the partially peritonealised organ surfaces (Figure 34). 

**Figure 35:**
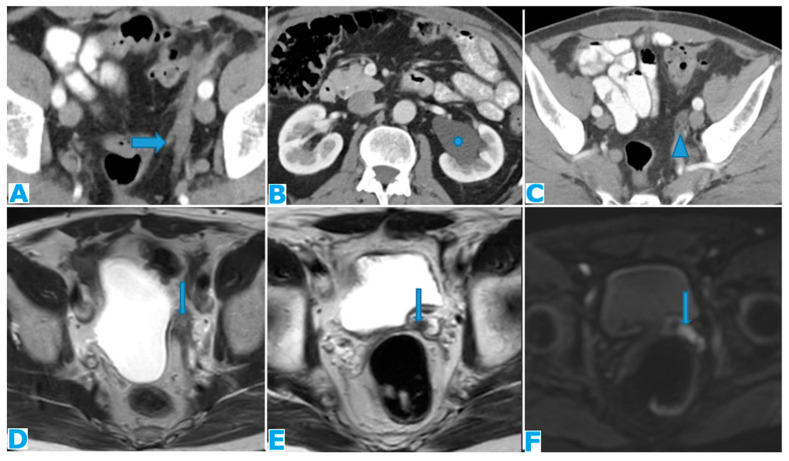
Axial CE-CT (**A**–**C**). Axial T2WI (**D**,**E**); DWI (**F**). PC from mucinous adenocarcinoma of the appendix: Notice the peritoneal deposit within the left lateral pelvis (arrow in (**A**)) as an elongated soft tissue mass. The patient presented with a left ureterohydronephrosis (* in (**B**)) due to the pelvic deposit, which obstructed the ureter (arrowhead in (**C**)). Paravesical spaces are peritoneal recesses that cover, on each side, the distal ureter, the seminal vesicle, and the deferent duct. Note the deposit within the left paravesical space and how it obstructs the left ureter (arrow in (**C**)). The deposit also follows the course of the left deferent duct (arrow in (**E**,**F**)).

As ureters are in close contact with the peritoneum at the paravesical spaces, pelvis deposits may be the cause of ureterohydronephrosis, and this is frequently overlooked, especially as the only sign of PC. Any deposit along the posterior parietal peritoneum in the proximity of the course of the ureter may also be the cause of a urinary obstruction, but the pelvis is a very frequent location (Figure 35).

Occlusion may occur as a result of pelvis deposits within the peritonealised portion of the rectum.

Another common site of PC in the pelvis is the ovaries. As seen previously, these are extraperitoneal organs, but are considered intraperitoneal, as they communicate with the peritoneal cavity. This is the reason why ovaries are included among the PC locations.

**Figure 36:**
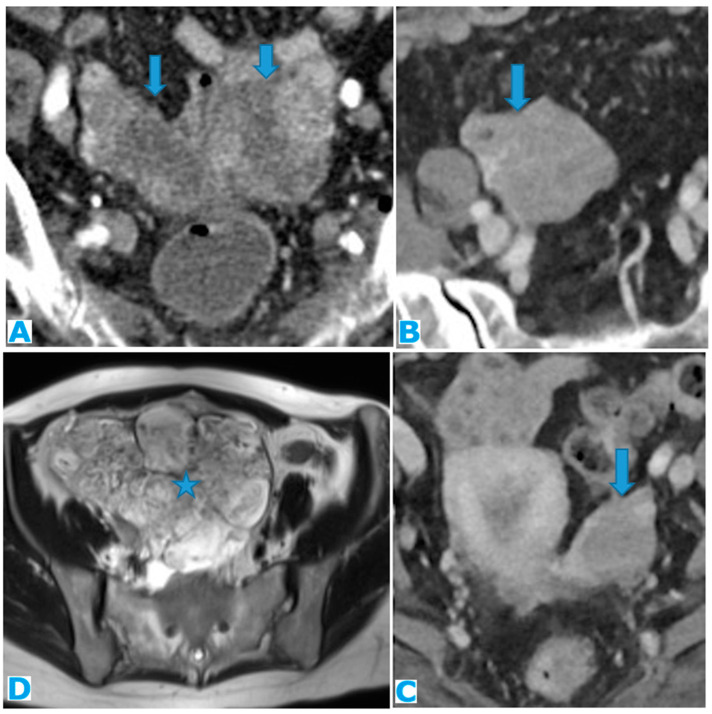
Axial CE-CT (**A**). PC from sigmoid adenocarcinoma: bilateral ovarian metastases as complex cystic masses with solid poles (arrows). Axial CE-CT (**B**,**D**). PC from colon adenocarcinoma: bilateral ovarian metastases as solid masses (arrow). Axial T2WI (**C**). PC from mucinous tumour of the appendix: left ovarian metastases (*) presenting as a predominantly hyperintense mass due to the mucin content.

Compared to primary ovarian tumours, ovarian metastases seem to be smaller and more frequently bilateral, showing more uniform cysts and more moderate enhancement of the solid portions [34]. However, a solid appearance may also be found, or even characteristics resembling the primary tumour (Figure 36).

The term Krukenberg tumour is sometimes misused in the setting of ovarian metastases from a gastrointestinal tumour, as its use should be limited to ovarian metastasis from a poorly differentiated adenocarcinoma with signet ring cell features [35]. Krukenberg tumour should be considered in the differential diagnosis when solid bilateral ovarian masses containing intratumoural cystic components are detected, even in the absence of a primary malignancy [36].

## 4. Peritoneal Fluid Circulation–Ascites

Now that the peritoneal anatomy has been revised, the focus is placed on the fluid contained in the peritoneal cavity, as it plays a determinant role in the seeding of the deposits.

Peritoneal fluid circulates following the path determined by ligaments and mesenteries, under the influence of the abdominal pressure fluctuations caused by respiration and intestinal peristalsis.

In the case of tumour spread to the peritoneum, the quantity of the peritoneal liquid rises due to two conditions: the obstruction of the lymphatics in charge of the resorption and the overproduction of fluid caused by the vascular permeability factor secreted by the tumour cells [37,38].

Fluid in the inframesocolic space naturally goes down on the right of the small bowel mesentery [39] through the mesenteric leaves, and on the left through the medial mesosigmoid, thus forming an area of arrested flow. It then reaches the pelvic recesses, the most gravity-dependent spaces. After filling the pelvis, it goes up the paracolic gutters: preferentially on the right, as the left gutter is shallower, and the flow is cranially limited by the phrenicocolic ligament [40] (Figure 1b). On the right, it reaches the subhepatic space and, finally, the right subphrenic space, where most of the peritoneal lymphatic clearance takes place, along with the omentum [40]. Therefore, places that normally constitute a free route or barrier for the flow or where most of the resorption takes place need to be particularly scrutinised.

Table 1 summarises the favoured locations for peritoneal seeding and the underlying reasons for this.

**Figure 37:**
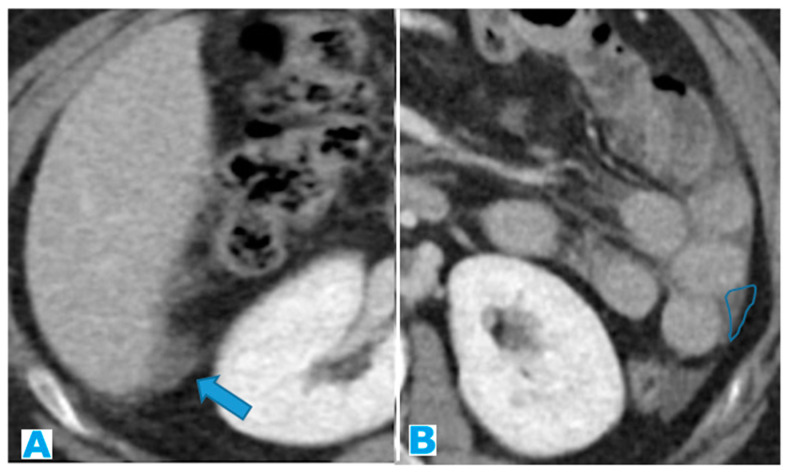
Axial CE-CT (**A**,**B**). PC from ovarian carcinoma: minimal ascites within the subhepatic space (arrow in (**A**)) and between the SB loops, triangle-shaped (**B**).

Ascites may be one of the first signs of PC. Its appearance correlates closely with its volume: if minimal, is only be found in the pelvic recesses, surrounding the liver and the spleen, or between the small bowel loops, in a triangle-shaped fashion (Figure 37). If quantity increases, it fills up the gutters, the omentum, and the mesentery leaves.

**Figure 38:**
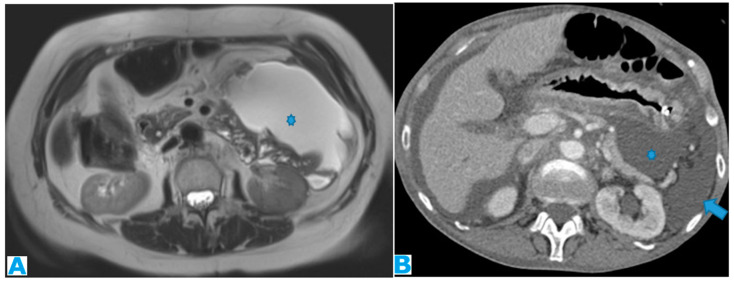
Axial T2WI (**A**). PC from ovarian adenocarcinoma: loculated ascites within the mesentery (*). Axial CE-CT (**B**). PC from breast carcinoma: concomitant ascites within greater (arrow) and lesser (*) sacs.

Suspicious signs may be ascites with rounded or bulging contours, or concomitantly present in the greater and lesser sacs (Figure 38).

**Figure 39:**
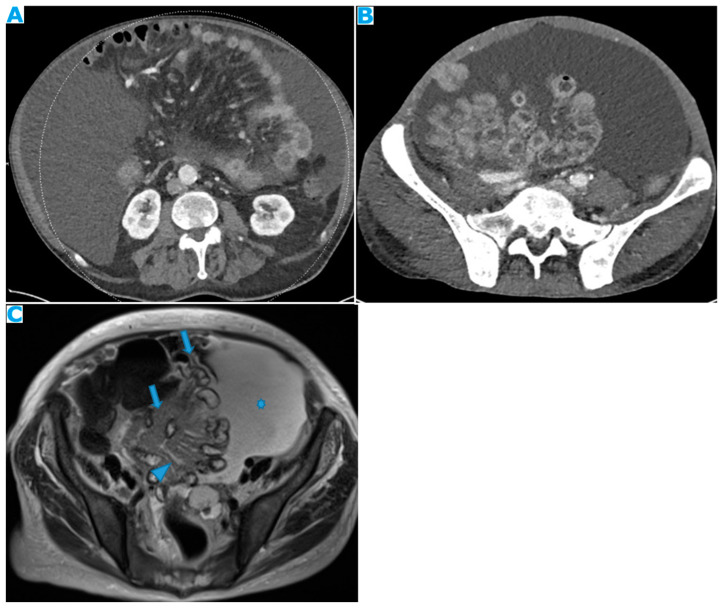
Axial CE-CT (**A**,**B**). (**A**): non-malignant ascites; (**B**): malignant ascites from colon adenocarcinoma. Observe how SB loops float freely in (**A**), with an anterior disposition. In a malignant ascites (**B**), SB loops are drawn posteriorly and lose contact with the anterior abdominal wall (tethered bowel sign) due to the rigid infiltrated mesenteric leaves. Axial T2WI (**C**). PC from endometrial carcinoma: diffuse mesenteric deposit seeding (arrow) that causes SB retraction (tethered bowel sign). Notice that, despite the massive ascites (*), there is little liquid between the rigid infiltrated mesenteric leaves (arrowhead).

Another important clue is how SB loops behave in peritoneal liquid: in nonneoplastic ascites or in the early stages of PC, they float freely, with an anterior location, whereas in advanced PC, as the mesentery becomes fibrotic and rigid, SB loops are pulled back centrally and posteriorly (the tethered bowel sign) [41]. Characteristically, there is little fluid between these rigid infiltrated mesenteric leaves, while fluid is predominant elsewhere in the peritoneal cavity (Figure 39).

**Figure 40:**
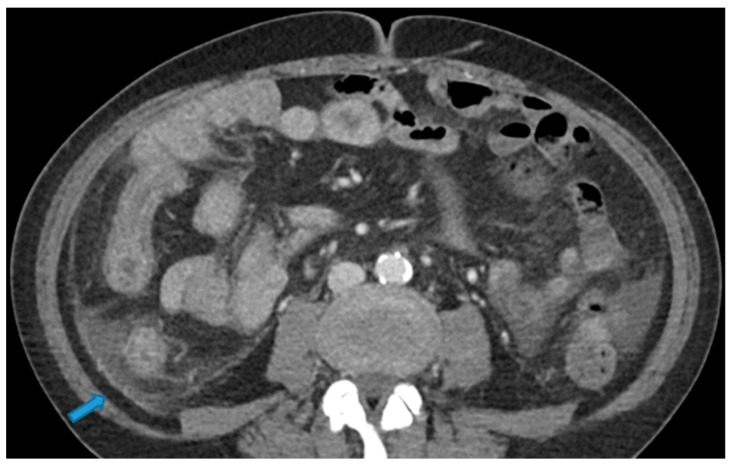
Axial CE-CT. Observe the enhancing pseudonodular parietal peritoneum (arrows), which corresponds to collaterals vessels, and is not to be mistaken for peritoneal deposits.

In the setting of portal hypertension and non-malignant ascites, collateral vessels within the parietal peritoneum should be cautiously noted, so as not to misdiagnose them as deposits (Figure 40).

## 5. Deposit Behaviour in Cross-Sectional Images

With regard to signal, the deposit does not fall far from the primary tumour; that is, deposits will usually show a density/signal intensity and enhancement pattern resembling the primary tumour, as extraperitoneal metastases do.

Thus, one should always bear in mind the underlying histology and imaging features of the primary tumour and contemplate the possibility of a second primary tumour if a discrepancy is found.

In addition, in the event of no known primary tumour, which occurs in about 3–5% of cases of PC [2], the behaviour in the different MR sequences and, to a lesser degree, in CT, may suggest the origin, despite the non-specificity of the imaging findings.

Indeed, knowledge of the underlying deposit content responsible for the appearance of high signal intensity in T1- and T2-weighted images of peritoneal deposits is an important diagnostic criterion that can contribute to the diagnosis of the primary tumour.

Table 2 summarises the behaviour of the peritoneal deposits according to their appearance on MR and CT, their content, and the corresponding primary tumour, regardless of cell line.

T1 hyperintensity may be observed within a deposit due to three contents: melanin, blood, and calcium.

**Figure 41:**
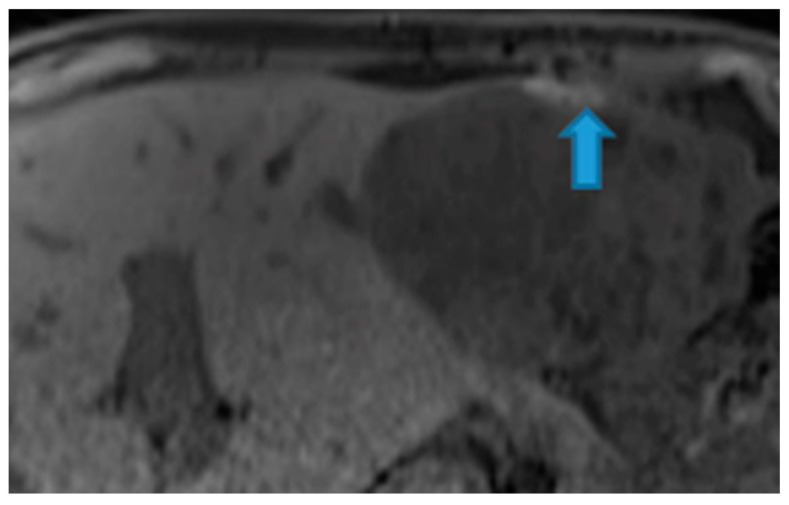
Axial NE FST1WI. PC from melanoma: Note the hyperintense melanin-containing perihepatic deposits (arrow).

**Figure 42:**
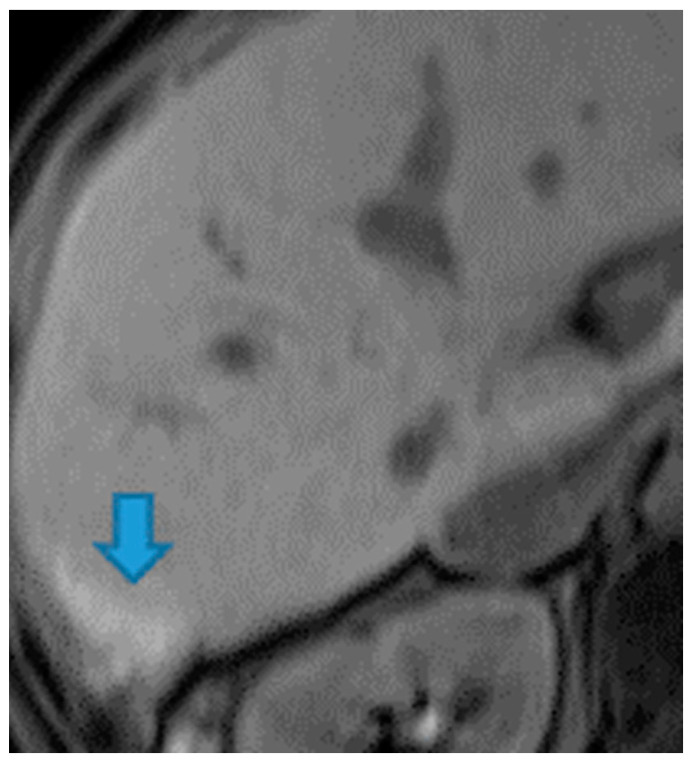
Axial NE FST1WI.PC from serous ovarian adenocarcinoma: Hyperintense perihepatic deposits (due to calcified content) (arrow).

**Figure 43:**
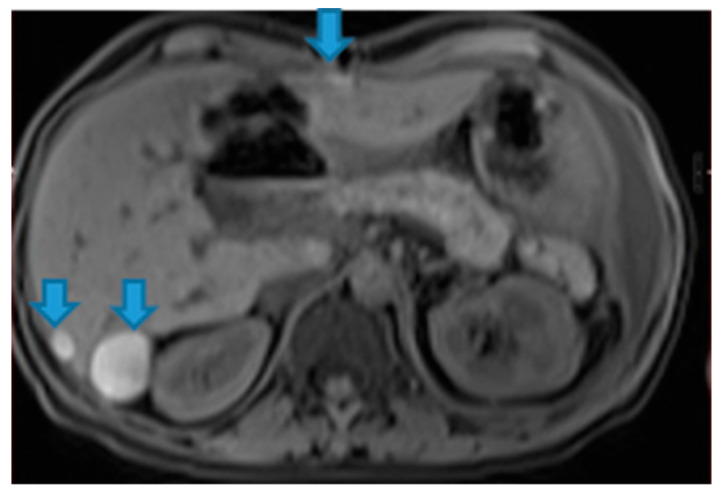
Axial NE FST1WI PC from serous ovarian adenocarcinoma: Blood-containing peri and subhepatic deposits (arrows).

- Melanin-containing deposits: from melanoma (Figure 41).

- Calcium-containing deposits: mucinous tumours of different origins (ovary, stomach, colon, pancreas, appendix, gallbladder, urachus) may calcify (Figure 42).

- Blood-containing deposits: blood content is frequently found in peritoneal deposits from hypervascular tumours of different origins (for instance, clear-cell and granulosa ovarian tumours) (Figure 43).

T2 hyperintensity can reflect different contents within deposits: myxoid, non-mineralised cartilage, or mucin. This may also be due to keratin in the setting of a squamous differentiation.

**Figure 44:**
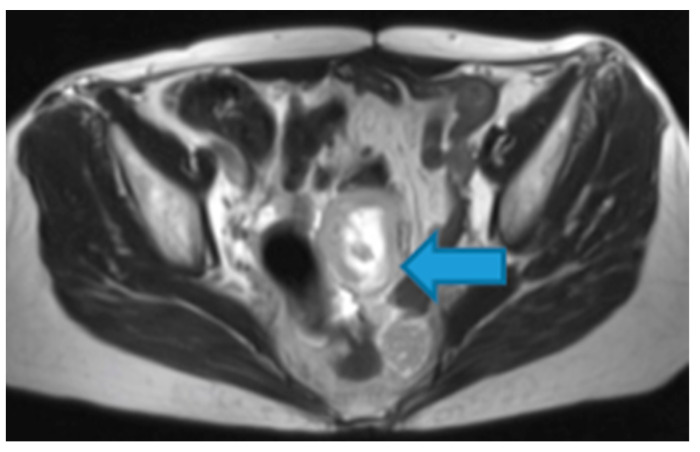
Axial T2WI. Myxoid leiomyosarcoma of the uterus: patient with pelvic deposits in the setting of a relapse (arrow). Observe the deposit central high SI on T2WI due to the myxoid content.

**Figure 45:**
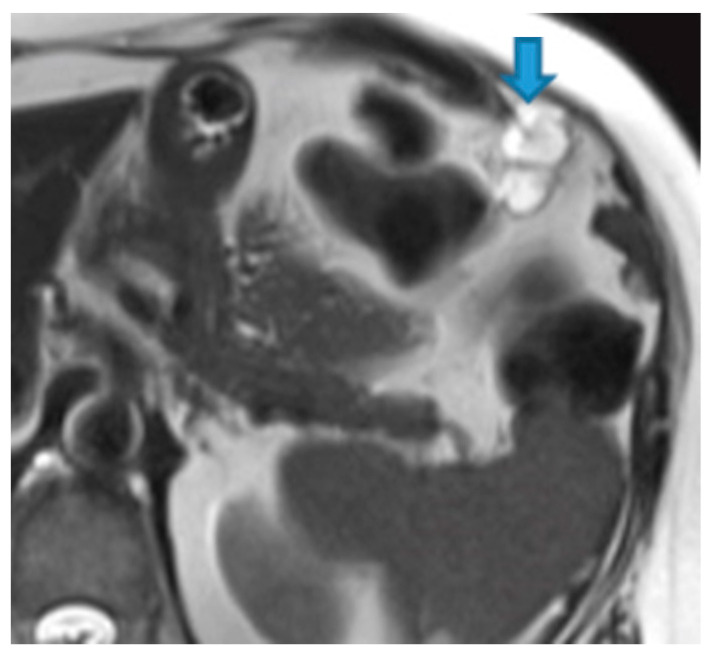
Axial T2WI. Peritoneal deposit from rib condrosarcoma: Omental deposit showing high SI on T2WI, due to the non-mineralized cartilage content (arrow).

**Figure 46:**
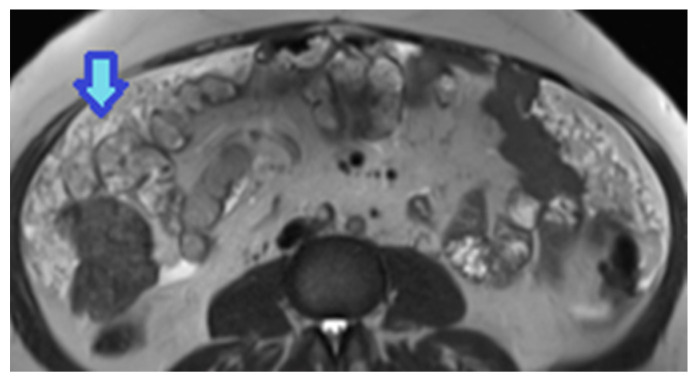
Axial T2WI. PC from mucinous adenocarcinoma of the urachus: Hyperintense omental-cake due to mucinous content (arrow).

**Figure 47:**
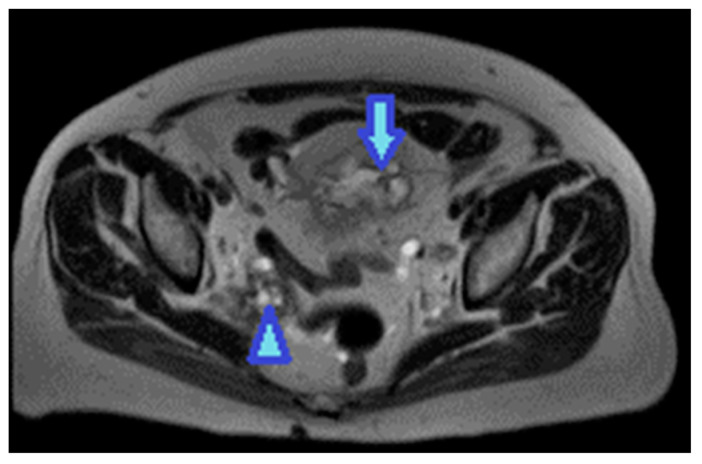
Axial T2WI. Bladder adenocarcinoma with a squamous differentiation. Observe the locally advanced vesical tumor (arrow). Note the right pelvic peritoneal deposit (arrowhead), showing the same imaging features as the tumour. The high signal on T2WI may be due to the keratin formed by the squamous differentiation.

- Myxoid-containing deposits: as in myxoid liposarcoma (Figure 44).

- Non-mineralised cartilage-containing deposits: from chondrosarcoma (Figure 45).

- Mucin-containing deposits: from mucinous tumours arising on different organs, namely ovary, stomach, colon, pancreas, appendix, gallbladder, and the urachus (Figure 46).

- Keratin-containing deposits: from tumours showing squamous differentiation (Figure 47).

**Figure 48:**
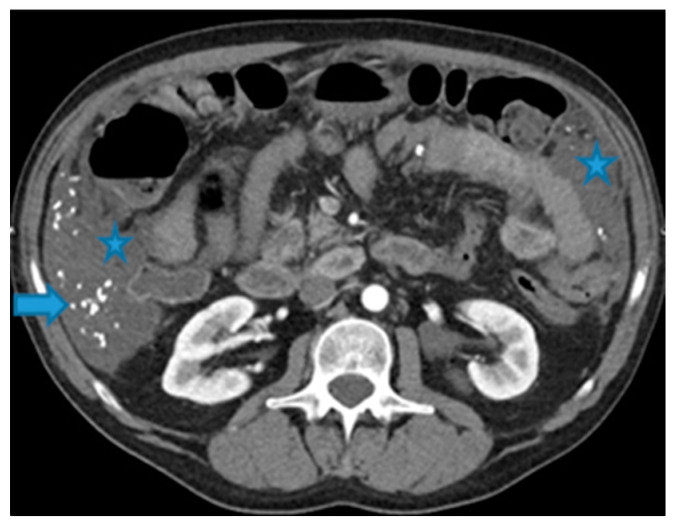
Axial CE CT. PC from colloid adenocarcinoma of the caecum: Observe the specks of calcification (arrows) scattered throughout the deposits (*).

CT hyperdensity can be due to calcium (Figure 48) or blood content. Calcification may occur secondary to treatment.

**Figure 49:**
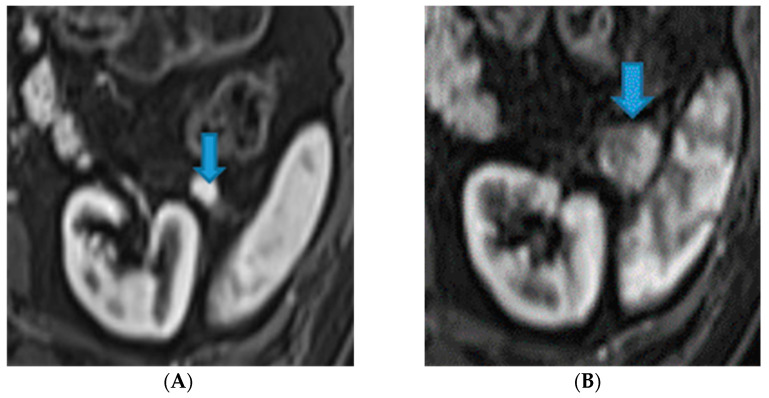
Axial CE portal phase FST1WI. PC from clear cell renal carcinoma: Note the hypervascular deposit adjacent to the spleen that was mistaken for an accessory spleen (arrow in (**A**)). Observe the growth on the follow-up CT (arrow in (**B**)).

**Figure 50:**
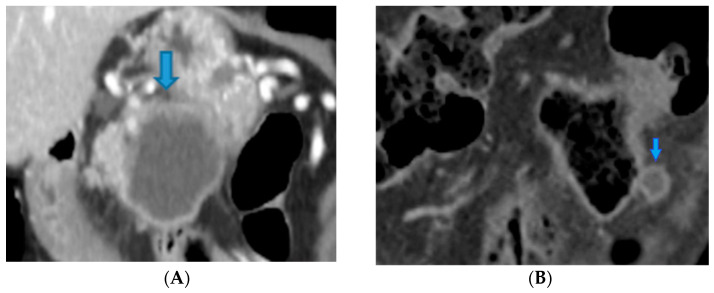
Coronal MPR CE CT PC from pancreatic cystoadenocarcinoma: Observe the primary tumour (arrow in (**A**)) and its deposit within the sigmoid mesocolon (arrow in (**B**)). Notice the hypovascular resemblance between them.

The vascular pattern of deposits also correlates with the characteristics of the primary tumour and may impact the differential diagnosis (Table 3) depending on whether deposits are hyper- (Figure 49) or hypovascular (Figure 50).

## 6. Differential Diagnosis

It is worth underlining that, in oncologic patients, not all peritoneal abnormalities correspond to carcinomatosis; hence, PC needs to be differentiated from other neoplastic and nonneoplastic conditions (inflammatory, infectious, and other types of benign causes, noninflammatory and noninfectious) (Table 4). This may be a challenging task as imaging features often overlap.

### 6.1. Inflammatory

#### 6.1.1. Omental Infarction—Figure S1

Omental infarction is a rare cause of acute abdomen, usually self-limiting, that presents with nonspecific symptoms. The right omentum is more commonly affected, as it moves more freely, and its vascularization is longer and more tortuous. It can be primary or secondary to other processes such as tumour, hernia, or postoperative adhesions [42].

It is usually a straightforward diagnosis of CT: striking fat stranding of the infarcted omentum with minimal or no involvement of the adjacent bowel (this is the reason why the fat stranding is usually described as disproportionate) [43].

Omental infarction may mimic PC at first glance, but the clinical setting and the inflamed aspect of the omental fat usually reveal the diagnosis. The importance of the correct diagnosis upon imaging relies on its conservative management unless superinfection occurs.

#### 6.1.2. Peritoneal Amyloidosis—Figure S2

Amyloidosis is a rare disease consisting of abnormal protein deposition throughout the body, which more frequently occurs in the gastrointestinal tract, kidneys, and heart. Peritoneal deposition is very rare and can mimic PC [44].

Two forms of peritoneal involvement have been described [45]: diffuse, in which the peritoneum is diffusely thickened, and nodular, where mesenteric masses are the key finding. Calcifications may be found within the deposits.

#### 6.1.3. Peritoneal Sarcoidosis—Figure S3

Sarcoidosis is a systemic granulomatous disease of unknown aetiology. The most common location is the lung [46]. Extrapulmonary involvement is found in 30% of all cases, with the abdomen being the most frequent site, and patients typically present with hepatosplenomegaly. Peritoneal involvement is a rare presentation, and the most frequent findings are ascites and peritoneal nodules. Peritoneal involvement is most frequently accompanied by a generalised disease that reveals the diagnosis, but a histological exam is necessary to confirm it [47].

#### 6.1.4. Familial Mediterranean Fever—Figure S4

This is a hereditary condition characterised by recurrent episodes of fever and systemic serosal inflammation, which mainly occurs in the abdomen. It especially affects patients of Mediterranean heritage. Imaging features are nonspecific; if it presents with ascites and peritonitis, it may strongly mimic PC [48]. The most fatal complication of FMF is amyloidosis and the chances of developing it are higher if left untreated [49].

#### 6.1.5. Encapsulating Sclerosing Peritonitis—Figure S5

This is a rare but serious condition. It can be idiopathic or secondary, either to peritoneal dialysis or other causes, benign (such as surgery, peritonitis, cirrhosis, and enteritis) and malignant (such as pancreatic and renal adenocarcinoma) [50]. It presents with recurrent episodes of bowel obstruction caused by a thickened and calcified peritoneal membrane that encircles the bowel [50].

### 6.2. Infectious

#### 6.2.1. Peritoneal Tuberculosis—Figure S6

Peritoneal tuberculosis may be a challenging entity to diagnose, given its insidious onset, its vague clinical presentation (abdominal pain and distention, weight loss, and fever), and the difficult isolation of the agents (*M. tuberculosis* complex) [51].

It is important to achieve a prompt diagnosis, as a delay in treatment may cause a worse outcome.

Imaging classification includes three main types [52]:

- Wet (the most common), where the salient feature is ascites, either free or loculated, which may show high attenuation on CT due to the high protein contents.
- Dry, where cellular content is predominant.
- Fibrotic fixed, where the main features are fibrotic changes, causing clustered SB loops.
- An in-between state may also be found (fibrotic mixed).

The findings may overlap with PC but the presence of a smooth peritoneum with minimal thickening and pronounced enhancement suggests PT, whereas nodular implants and irregular peritoneal thickening suggest PC. Prominent mesenteric and retroperitoneal adenopathies may be seen in PT, which may present with a necrotic centre and rim enhancement.

#### 6.2.2. Peritoneal Echinococcosis—Figures S7 and S8

This is a parasitic condition secondary to the peritoneal seeding of Equinococcus larvae. The two main species of the Echinococcus tapeworm are *E. granulosus*, the causal agent in cystic echinococcosis, also known as hydatid disease hosts, and *E. multilocularis*, in alveolar echinococcosis [53]. Both domestic and wild canines are the definite hosts and humans are potentially involved as intermediate hosts [54]. In both entities, the initial infestation is localised to the liver.

*E. granulosus* hydatid cysts appear as well-defined, thick-walled cystic lesions, with mural calcifications as a characteristic feature [55]. Peritoneal involvement generally results from the rupture of hepatic or splenic cysts, with ascites and peritoneal enhancement as the main imaging features. Daughter cysts scattered throughout the peritoneal cavity may also be found.

Hepatic alveolar echinococcosis usually appears as a tumour-like infiltrative and partially calcified heterogeneous mass, with cystic and necrotic components. It may spread directly to the peritoneum, resulting in peritoneal lesions presenting the same imaging characteristics that inevitably mimic features of peritoneal carcinomatosis. The most salient feature is the infiltrative behaviour, with a tendency to invade adjacent structures. It is important to achieve an early diagnosis, as complete surgical excision is the only curative treatment [56].

### 6.3. Benign Noninflammatory/Noninfectious

#### 6.3.1. Splenosis/Accessory Spleen—Figure S9

These terms comprehend ectopic foci of splenic tissue that may be found throughout the abdominal cavity, differing in whether the cause is acquired (splenosis) or congenital (accessory spleen).

Splenosis is usually a consequence of spleen injury, either from trauma or surgery, resulting in splenic fragments that acquire a vascular supply and may grow. They may seed on the liver, as in the case shown in the figure, although they most frequently seed in the left lobe, with a subcapsular location. Intrahepatic splenosis usually shows increased enhancement on the arterial phase and may be a potential pitfall for hepatocarcinoma, neuroendocrine metastases, or adenoma [57,58,59]. Definitive diagnosis is confirmed using heat denaturation red blood cell scintigraphy.

Accessory spleens enhance similar to the spleen, although this may be difficult to evaluate due to their small size. As a rule, these nodules are well-defined and homogeneous, whereas peritoneal deposits may tend to be more irregular and heterogeneous.

#### 6.3.2. Foreign Body Bowel Perforation—Figure S10

Inflammatory–phlegmonous changes secondary to bowel perforation caused by a foreign body may mimic peritoneal carcinomatosis in oncologic patients.

#### 6.3.3. Encapsulated Omental Fat Necrosis—Figure S11

Either spontaneous or secondary to inflammation or trauma, it is usually asymptomatic and its presentation on imaging depends on the time of evolution. Using CT, if acute, it appears as an omental or mesenteric focal fat stranding that may have a discrete mass effect on the adjacent organs; over time, it shrinks and becomes well-defined and peripherally calcified. Initially, it may mimic a liposarcoma and, when it becomes fibrotic (usually, it shows heterogeneous low signal intensity in T1 and T2WI and may slightly enhance), it can be misdiagnosed as a deposit, especially in oncologic patients. Clinical history and previous imaging examinations are essential to achieve a correct diagnosis.

#### 6.3.4. Endometriosis—Figure S12

This is a benign condition characterised by the implantation of endometrial tissue outside the uterine cavity. Peritoneal endometriosis is usually accompanied by other imaging findings that suggest the diagnosis; however, peritoneal involvement may appear as the sole finding. Endometriotic deposits will likely show areas of high signal intensity in T1WI due to blood content and/or low signal intensity in T2WI due to fibrosis.

#### 6.3.5. Leiomyomatosis Peritonealis—Figure S13

This is a rare condition that occurs in women of reproductive age, characterised by the development of multiple leiomyomas within the peritoneum. Potential risk factors are increased levels of endogenous/exogenous oestrogens [60] and prior laparoscopic myomectomy [61].

Malignant transformation is a rare complication [62]. Peritoneal leiomyomas are observed as iso-hypodense nodules using CT with a muscle-like signal intensity using MR and strong homogeneous enhancement, which becomes heterogeneous (due to necrosis and haemorrhage) in the event of malignant transformation. This entity may mimic a PC of unknown origin. Definitive diagnosis is made histopathologically.

#### 6.3.6. Desmoid Tumours—Figure S14

Desmoid tumours (DT) belong to a heterogeneous group of locally aggressive fibromatosis, although non-metastasising, that may arise throughout the body, most commonly extra-abdominally. In 30% of patients, there is an association with familial adenomatous polyposis and, in this setting, tumours are most frequently multiple and intra-abdominal (80% compared to 5% of intra-abdominal sporadic DT) [63].

There is also an association with pregnancy and prior trauma and surgery have been described as possible risk factors [64]. In the abdomen, they are mostly mesenteric, although pregnancy-associated DTs usually occur within the abdominal wall [65].

DTs are soft-tissue masses that may show either well-demarcated or ill-defined margins that extend into the adjacent mesenteric fat. They are usually isodense to muscle; their signal intensity on MR depends on the predominant content (myxoid, cellular, or fibrosis). Enhancement ranges from moderate to intense. As infiltrating mesenteric soft-tissue masses, they can mimic PC.

### 6.4. Malignant

#### 6.4.1. Primary Peritoneal Serous Carcinoma—Figure S15

It occurs in women, predominantly postmenopausal, and is challenging to diagnose as it resembles advanced epithelial ovarian carcinoma (AEOC), both in imaging and in the histological exam. It occurs less frequently than AEOC and has a worse prognosis [66]. The distinguishing features are the sparing of the ovaries or the disproportionate burden of extra-ovarian disease compared to the ovarian involvement [67].

#### 6.4.2. Pseudomyxoma Peritonei (PMP)—Figure S16

This clinical term refers to a syndrome characterised by the presence of mucinous loculated ascites within the peritoneum [68].

These mucinous septated collections disseminate along the peritoneal surface and their mass effect causes a scalloped appearance of adjacent organs. Pseudomyxoma cells lack adherence molecules on their surface, so they spread by a redistribution phenomenon, which means they follow the current of the intraperitoneal fluid and tend to accumulate at the gravity-dependent and resorption sites described at the beginning of this review [69]. Thus, the mobile small bowel is initially spared thanks to its continuous peristaltic movement.

This entity has been classically associated with a perforated epithelial neoplasm of the appendix; although less frequently, it can originate from mucinous tumours arising from other organs [70]. It should be distinguished from mucinous peritoneal carcinomatosis—as these conditions differ histologically—upon imaging findings and prognosis [71].

#### 6.4.3. Peritoneal Malignant Mesothelioma (PMM)—Figure S17

This very rare and fatal primary malignancy of the peritoneum shows features that overlap those of PC; thus, it is difficult to distinguish these entities using imaging alone. The link with asbestos exposure is weaker than in pleural mesothelioma but remains its best-defined risk factor [72]: a history of asbestos exposure or the presence of pleural plaques can be helpful in differentiating PMM from PC.

#### 6.4.4. Desmoplastic Small Round Cell Tumour—Figure S18

A rare mesenchymal malignancy that arises from peritoneal surfaces as multiple soft-tissue masses. This occurs most frequently in young male patients. Imaging features overlap with other peritoneal malignant tumours. Calcification may occur on 30% of the cases [73].

#### 6.4.5. Peritoneal Lymphomatosis and Peritoneal Sarcomatosis—Figures S19 and S20

As described at the beginning of this review, the term peritoneal carcinomatosis is used when the disease is caused by epithelial cells. The peritoneum may also be the soil of malignant nonepithelial cellular lines, mesenchymal or lymphoid, and thus refer to sarcomatosis and lymphomatosis, respectively [26].

In the setting of an unknown primary tumour, the differential diagnosis should always include the possibility of another cell line being the origin of the peritoneal deposits.

Peritoneal lymphomatosis (PL) is a rare condition but important to suspect, as it responds favourably to chemotherapy. It is uncommon, as the peritoneum lacks lymphoid tissue, and the underlying mechanism is unknown. It is associated most frequently with diffuse large B-cell lymphoma, although it can occur in many subtypes [74].

Imaging features include homogeneous soft-tissue diffuse infiltration of peritoneal leaves [26], associated with prominent retroperitoneal lymph nodes [75] and bulky mesenteric lymph nodes that surround the mesenteric vessels and the perivascular fat on both sides (sandwich sign) [76]. In the presence of lymph nodes, diagnosis is easier, as the burden of the nodal disease is usually disproportionate to the peritoneal disease and its distribution is more diffuse than in PC, where lymph nodes are usually located adjacent to the primary tumour [77].

Ascites, which can be massive in PC, is rather mild in PL, and hepatosplenomegaly occurs more frequently.

Isolated peritoneal disease with no bowel or lymph node involvement is rare [78].

Bowel obstruction is uncommon, even in extensive lymphomatous infiltration of small bowel, due to its lack of desmoplastic reaction [79].

Peritoneal sarcomatosis (PS) is defined as a disseminated intraperitoneal spread either from an intra-abdominal primary sarcoma or from extremity sarcomas [80]. Sarcomatous peritoneal deposits tend to be larger, more hypervascular and heterogeneous (Table 2) than in PC. In addition, lymph node involvement is rare. Ascites is variable [80] and hemoperitoneum may occur more frequently than in PC [81].

Bowel obstruction and hydronephrosis tend to be more common in PC [80].

Despite these differences, the diagnosis of PL/PS based on imaging can be difficult to achieve as PC is much more frequent; thus, the diagnosis should be confirmed using histology.

## 7. Conclusions

To correctly diagnose PC, a systematic approach to the abdominal cavity is highly recommended. The knowledge of peritoneal anatomy and peritoneal fluid flow characteristics substantially contribute to an understanding of where to look for deposits and their appearance on cross-sectional imaging.

Indeed, in CT and MR, features of peritoneal deposits and their behaviour on contrast-enhanced cross-sectional images are related to the histologic characteristics of the primary tumour. Therefore, in the event of peritoneal carcinomatosis with unknown primary tumour, signal intensity/density characteristics of the peritoneal deposits, together with their vascular properties, may be helpful in the identification of the primary tumour.

Moreover, it can occur that the sole presenting sign of peritoneal carcinomatosis is intra-abdominal complications, such as bowel obstruction or ureterohydronephrosis, which should be considered suspicious in oncologic patients, even in the absence of clear radiological evidence of peritoneal deposits.

Furthermore, there are several benign and malignant peritoneal conditions that may mimic peritoneal carcinomatosis. Thus, even in oncologic patients, it is important to consider these conditions in the differential diagnosis with peritoneal carcinomatosis. However, when relying solely on imaging, it remains difficult to conduct a differential diagnosis, since the imaging features overlap between benign/other malignant conditions.

## Figures and Tables

**Table 1:** Favoured PC sites and underlying reasons.

Favoured PC Sites	Underlying Reason
Ileocecal region	Anchor of the small bowel mesentery
Sigmoid mesocolon	Area of arrested flow
Right paracolic gutter	Major gravity dependent pathway
Subhepatic space	Gravity dependence
Right subphrenic space and omentum	Resorption sites

**Table 2:** Behaviour of peritoneal deposits according to their appearance on MR and CT, their content and the corresponding primary tumour, regardless of the cell line.

Content	T1	T2	CT	Primary Tumour
Melanin	↑	↓	↓	Melanoma
Calcium	↑=	↓=	↑	Mucinous tumors (ovary, stomach, colon, pancreas, appendix, gallbladder, urachus) Serous papillary ovarian tumour
Blood	↑ ↑	↓ ↑	↑	Hypervascular tumours. High-grade ovarian tumours (serous and endometrioid adenocarcinoma). Clear cell ovarian carcinoma Granulosa cell tumour. In the subacute stage of a haematoma, the methemoglobin causes a high SI on T1WI, and a variable SI on T2WI (low in early subacute stage, high in late subacute stage).
Myxoid	↓	↑	↓	Myxoid tumours
Non mineralized cartilage	↓	↑	↓	Condrosarcoma
Mucin	↓	↑	↓	Mucinous tumours (ovary, stomach, colon, pancreas, appendix, gallbladder, urachus)
Keratin	↓	↑	↓	Squamous differentiation

↑: High signal intensity (SI)/attenuation, ↓: Low SI/attenuation, =: Isointense

**Table 3:** Relation between vascular pattern of the deposits and differential diagnosis of possible primary tumours.

Hypervascular Deposits	Hypovascular Deposits
Ovarian (clear cell, granulosa)	Mucinous tumours (ovary, stomach, colon, pancreas, appendix, gallbladder, urachus)
Breast carcinoma	Pancreas adenocarcinoma
Lung carcinoma	Liposarcoma (myxoid or undifferentiated)
Melanoma	
Sarcoma: - GIST - Leiomyosarcoma - Fibrous solitary tumour	
Renal cell carcinoma	
Neuroendocrine tumours	
Hepatocellular carcinoma	
Thyroid carcinoma	
Paraganglioma	
Choriocarcinoma	

**Table 4:** Differential diagnosis.

Inflammatory	Infectious	Benign Noninflammatory Noninfectious	Malignant
Omental infarction	Peritoneal tuberculosis	Splenosis Accessory spleen	Primary peritoneal serous carcinoma
Peritoneal amyloidosis	Peritoneal echinococcosis	Bowel perforation	Pseudomyxoma peritonei
Peritoneal sarcoidosis		Encapsulated omental fat necrosis	Peritoneal malignant mesothelioma
Familial Mediterranean fever		Endometriosis	Desmoplastic small round cell tumour
Encapsulated sclerosing peritonitis		Leiomyomatosis peritonealis	Peritoneal lymphomatosis
		Desmoid tumours	Peritoneal sarcomatosis

## Data Availability

Not applicable.

## Supplementary Materials

The following supporting information can be downloaded at: https://www.mdpi.com/article/10.3390/diagnostics13132253/s1. Figure S1. Omental infarction. Figure S2. Peritoneal amyloidosis. Figure S3. Peritoneal sarcoidosis. Figure S4. Familial Mediterranean fever. Figure S5. Encapsulated sclerosing peritonitis. Figure S6. Peritoneal tuberculosis. Figure S7. Peritoneal echinococcosis (same patient as Figure S58). Figure S8. Peritoneal echinococcosis. Figure S9. Accessory spleen. Figure S10. Bowel perforation. Figure S11. Encapsulated omental fat necrosis. Figure S12. Endometriosis. Figure S13. Leiomyomatosis peritonealis. Figure S14. Desmoid tumours. Figure S15. Primary peritoneal serous carcinoma. Figure S16. Pseudomyxoma peritonei. Figure S17. Peritoneal mesothelioma. Figure S18. Desmoplastic small round cell tumour. Figure S19. Peritoneal lymphomatosis. Figure S20. Peritoneal sarcomatosis.
