# Supplementary material for: Unravelling Peritoneal Carcinomatosis Using Cross-Sectional Imaging Modalities
Source: Diagnostics (Basel). 2023 Jul 3;13(13):2253. doi: 10.3390/diagnostics13132253 (PMC10340753; doi:10.3390/diagnostics13132253)
Supplement: Supplementary file 1 [file diagnostics-13-02253-s001.zip › diagnostics-2412560-supplementary.pdf]

Inflammatory:

Figures S1: Omental infarction

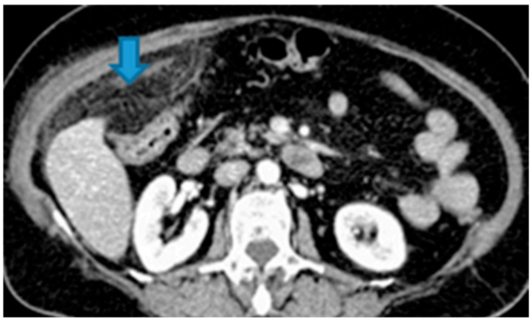

Patient with no known prior history, who presented with abdominal pain. Notice the salient fat stranding of the right omentum with mass effect on transverse colon but otherwise no involvement of the adjacent bowel otherwise. The diagnosis was omental infarction and it resolved within days.

Figure S2: Peritoneal amyloidosis

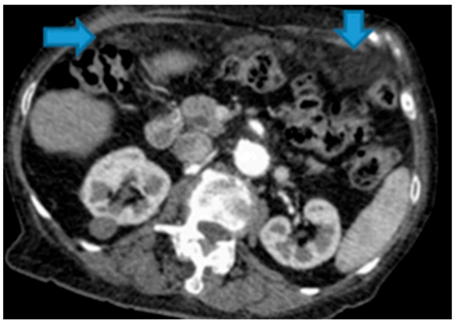

Axial CE-CT. Patient with known cardiac amyloidosis. Observe the soft-tissue nodular-pattern infiltration within the omentum. Findings were stable compared to a CT from 10 years prior. The peritoneal lesions were biopsied, and the histopathological exam concluded peritoneal amyloidosis.

Figure S3: Peritoneal sarcoidosis

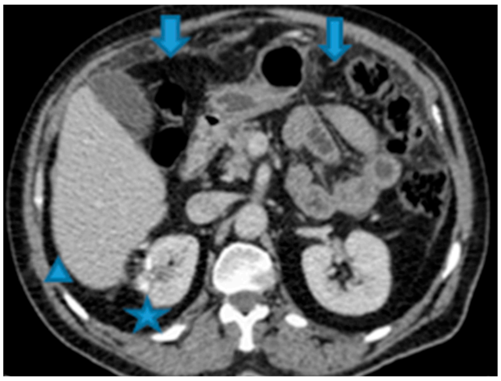

Axial CE-CT. Patient on surveillance for a low-grade papillary renal cell carcinoma who underwent surgery five years ago (*). Note the omental infiltration (arrows) and the nodular thickening of the hepatic surface (arrowheads). PC was first suspected, although warily, given its unlikeliness in the setting of a low-grade papillary renal cell carcinoma. A thorax CE-CT, not shown, revealed mediastinal adenopathies, and peritoneal biopsy proved this to be peritoneal sarcoidosis.

Figure S4: Familial Mediterranean fever

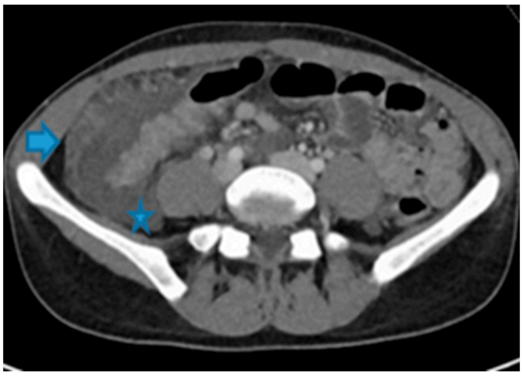

Axial CE-CT. A 25-year-old patient with no medical history presenting with abdominal discomfort. CT showed a peritoneal mass resembling an omental cake (arrows) with ascites (*) and bilateral pleural effusion (not shown). A PC of unknown primary tumour was suspected. Her referring doctor later provided further clinical information: the patient suffered from recurrent inflammatory episodes. She responded to oral colchicine and was diagnosed with familial Mediterranean fever.

Figure S5: Encapsulated sclerosing peritonitis

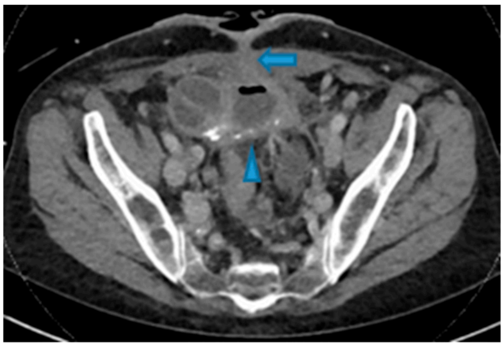

Axial CE-CT. Patient with a known peritoneal pseudomyxoma treated with surgery and CHIP who developed an encapsulating sclerosing peritonitis. Observe the clustered SB loops encircled by a thick calcified membrane (arrowhead). Note enterocutaneous fistula as a complication (arrow).

Infectious:

Figure S6: Peritoneal tuberculosis

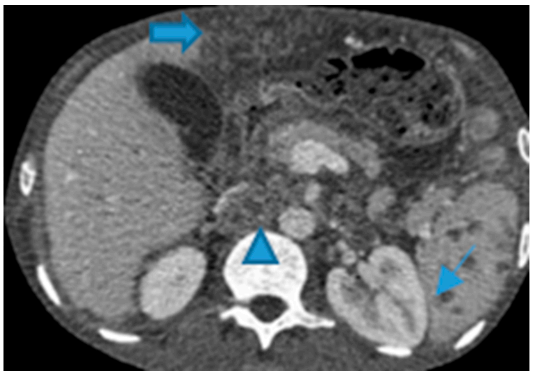

Axial CE-CT. Patient from Mali with a constitutional syndrome. CT showed multiple low density omental nodules (arrow) and retroperitoneal and mesenteric adenopathies (arrowhead). Multiple hypodense splenic lesions were also noted (fine arrow). Loculated subhepatic ascites and mild free ascites were observed (not shown). Thorax CT (not included here) also showed sternal lytic lesions with soft-tissue components, mediastinal adenopathies and bilateral pleural effusion. Differential diagnosis included disseminated tuberculosis and PC of unknown origin. PCR test of sample obtained by fine needle aspiration on an axillary adenopathy concluded disseminated tuberculosis (probably dry type, as the cellular content is the salient feature).

Figure S7: Peritoneal echinococcosis (same patient as Figure S8)

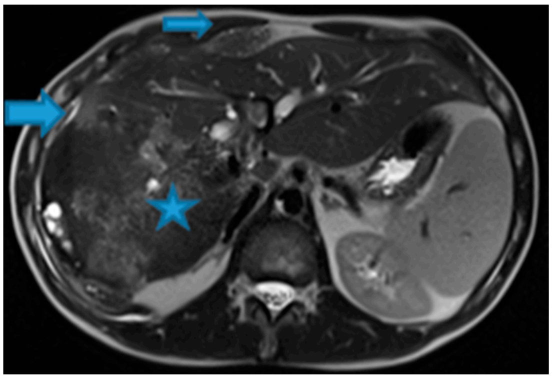

Axial T2WI. Patient with no prior history who presented to the ER with abdominal pain. A rectal mass (arrowhead) was discovered along with a mass in the liver (*) and several peritoneal nodules (arrow). All the lesions showed the same heterogeneous signal intensity and infiltrative behaviour. The rectal mass was endoscopically biopsied and a histology exam concluded it was an Echinococcus granulosus hepatic abscess with peritoneal dissemination.

Figure S8: Peritoneal echinococcosis

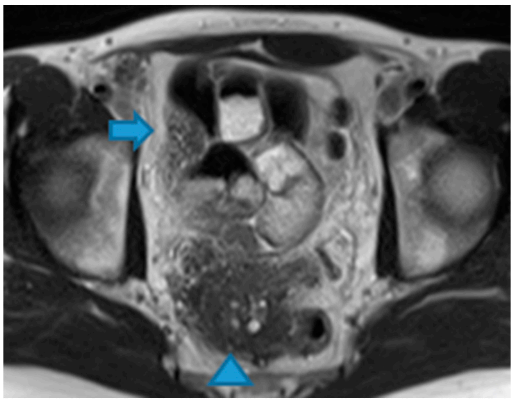

Benign non-inflammatory non-infectious:

Figure S9: Accessory spleen

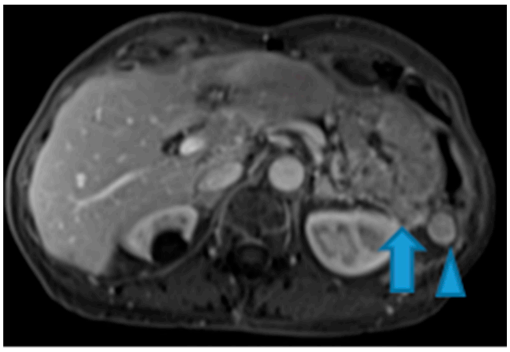

Axial CE portal phase FST1WI. Patient on follow-up for ovarian carcinoma. Left subphrenic deposits (arrow) that were mistaken for accessory spleens (the patient had undergone tumour resection, which included a splenectomy). Note the accessory spleen adjacent to the deposits (arrowhead) and the distinct differences between them: deposits show irregular contours and heterogeneous enhancement opposed to the smoothly outlined and homogeneously enhanced accessory spleen.

Figure S10: Bowel perforation

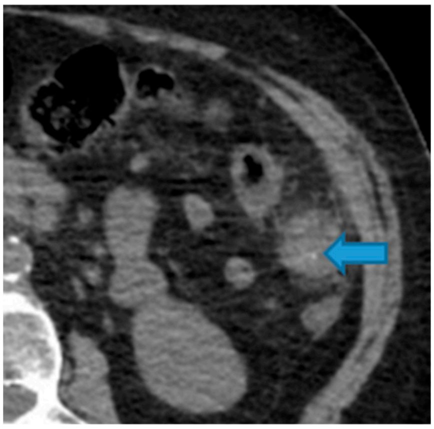

Axial NE-CT. Patient on follow-up for a breast carcinoma. A suspiciously positive lesion within the transverse mesocolon was noted on PET-CT (not shown) and the report concluded possible PC, given the oncologic history. When the CT was reviewed, a calcified central elongated foreign body was identified (arrow). The diagnosis shifted to a colon perforation due to a fish bone and resolved spontaneously.

Figure S11: Encapsulated omental fat necrosis

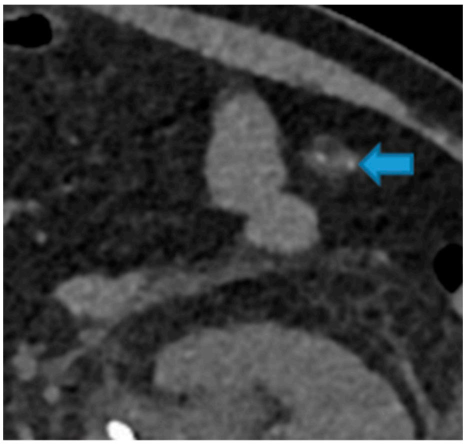

Axial NE-CT. Encapsulated omental fat necrosis (arrow) that was misdiagnosed as a peritoneal deposit on an ovarian malignancy follow-up. When compared to previous CT, it was stable and punctate calcifications were noticed within (arrow).

Figure S13: Leiomyomatosis peritonealis

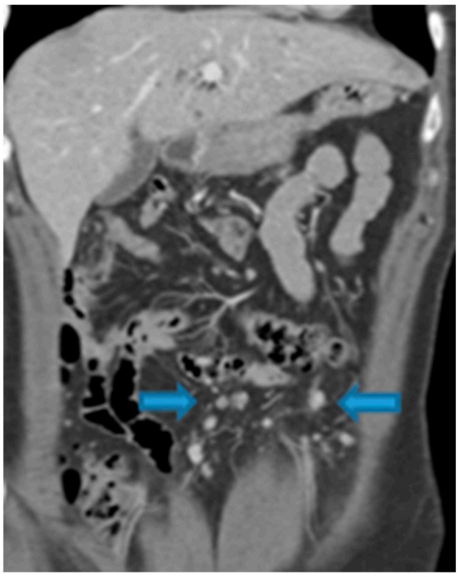

CE-CT coronal MPR. A 45-year-old patient with no known primary tumour. Peritoneal nodules were identified on a pelvic MR for uterine fibroids (not shown), and an abdominal MR was performed. Multiple hyperenhancing omental nodules were noted (arrows) and she was diagnosed with a PC of unknown origin. She underwent an explorative laparoscopy with partial omentectomy: it turned out to be a disseminated peritoneal leiomyomatosis that responded well to hormonal treatment.

Figure S12: Endometriosis

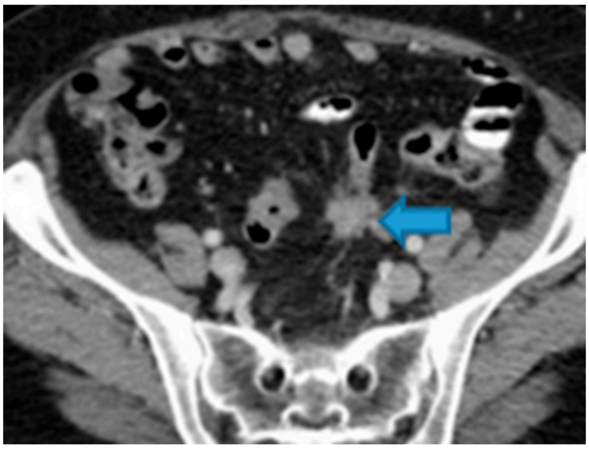

Axial CE CT. A 40-year-old female patient with no prior history who was incidentally discovered to have a spiculated lesion within the sigmoid mesocolon. The differential diagnosis included a peritoneal deposit of an unknown primary tumour or a desmoplastic reaction of an unknown neuroendocrine tumour. Biopsy revealed an endometriotic peritoneal deposit.

Figure S14: Desmoid tumours

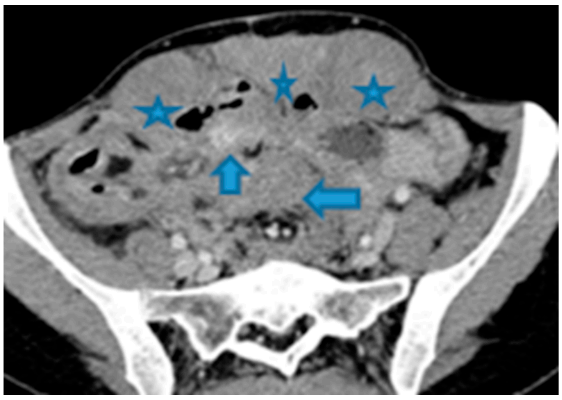

Axial CE-CT. Young female presenting with multiple desmoid tumours in the setting of Gardner´s syndrome: mesenteric (arrows) and within the anterior abdominal wall (*).

Malignant:

Figure S15: Primary peritoneal serous carcinoma

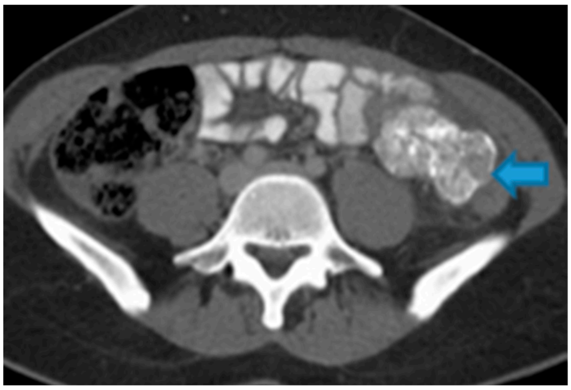

Axial CE-CT. Young female patient who presented with bloating. Note the extensive peritoneal disease (arrow), partly calcified. Calcifications suggest a mucinous tumour and, more frequently, a serous ovarian adenocarcinoma, but the ovaries (not shown) appeared almost normal. Thus, a primary peritoneal serous carcinoma was the first hypothesis and was proven by biopsy following a laparoscopic exam.

Figure S16: Pseudomyxoma peritonei

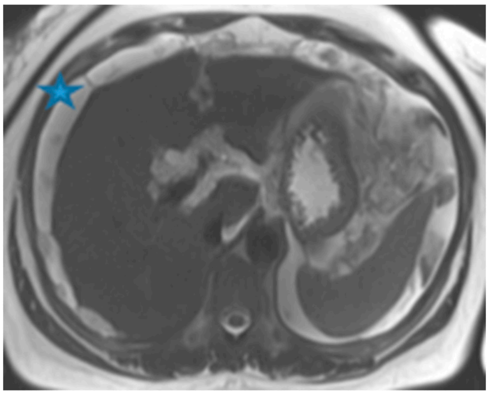

Axial T2WI. Observe loculated mucin (*) in this patient with a known PMP; notice the characteristic scalloped appearance of the coated organs.

Figure S17: Peritoneal mesothelioma

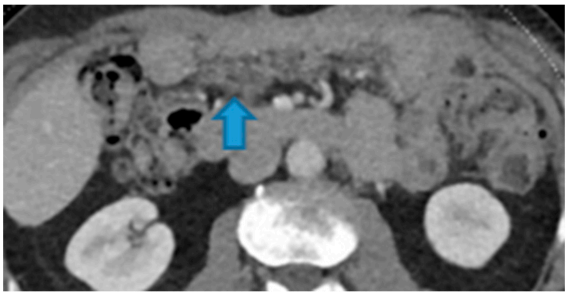

Axial CE-CT. Note deposits within the mesentery in this patient with a known PMM. It is difficult to distinguish PC from PMM on imaging alone. However, an asbestos exposure history or the presence of pleural plaques, as was the case there (not shown), could be helpful in differentiating PMM from PC.

Figure S18: Desmoplastic small round cell tumour

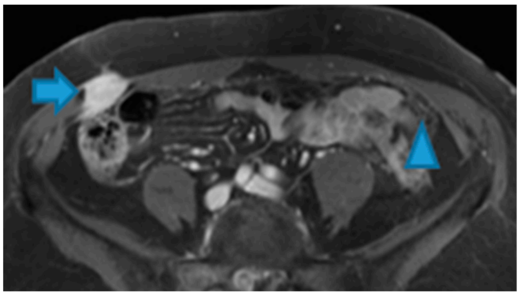

Axial CE portal phase FST1WI. A 20-year-old male patient with no medical record who presented with a palpable abdominal mass. Observe the mass within the anterior parietal peritoneum (arrow) with invasion of the abdominal wall muscles. Note the omental infiltration (arrowhead). The histological exam proved it was a desmoplastic small round cell tumour.

Figure S19: Peritoneal lymphomatosis

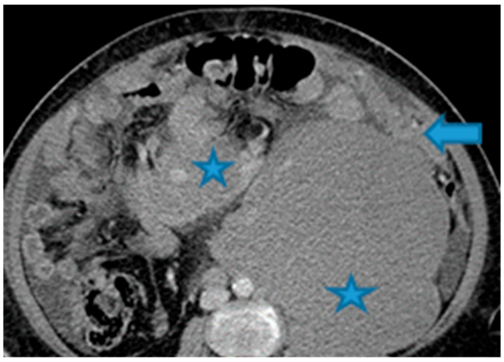

Axial CE-CT. Follicular NH lymphoma. Note the omental cake (arrow), which is suspicious for PC. Nevertheless, the presence of coexisting bulky retroperitoneal and mesenteric adenopathies (*) are imaging features that favour PL over PC.

Figure S20: Peritoneal sarcomatosis

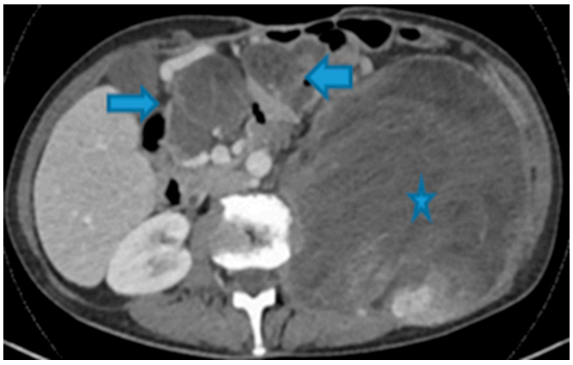

Axial CE portal phase FST1WI. Sarcomatosis from retroperitoneal liposarcoma. Patient who underwent surgery for the primary tumour and presented with a relapse showing a left retroperitoneal mass (*) and mesenteric deposits (arrows).
